# Ecosystem approach to harvesting in the Arctic: Walking the tightrope between exploitation and conservation in the Barents Sea

**DOI:** 10.1007/s13280-021-01616-9

**Published:** 2021-09-03

**Authors:** Michael R. Heath, Déborah Benkort, Andrew S. Brierley, Ute Daewel, Jack H. Laverick, Roland Proud, Douglas C. Speirs

**Affiliations:** 1 Department of Mathematics and Statistics, University of Strathclyde, Livingstone Tower, 26 Richmond Street, Glasgow, G1 1XH Scotland, UK; 2 Institute for Coastal Systems - Analysis and Modelling, Helmholtz-Zentrum Hereon, Max-Planck-Str. 1, 21502 Geesthacht, Germany; 3 Pelagic Ecology Research Group, Scottish Oceans Institute, Gatty Marine Laboratory, School of Biology, University of St Andrews, Fife, KY16 8LB Scotland, UK

**Keywords:** Acoustic data, Chlorophyll, Climate change, Ecosystem model, Fishing, Food web

## Abstract

Projecting the consequences of warming and sea-ice loss for Arctic marine food web and fisheries is challenging due to the intricate relationships between biology and ice. We used StrathE2EPolar, an end-to-end (microbes-to-megafauna) food web model incorporating ice-dependencies to simulate climate-fisheries interactions in the Barents Sea. The model was driven by output from the NEMO-MEDUSA earth system model, assuming RCP 8.5 atmospheric forcing. The Barents Sea was projected to be > 95% ice-free all year-round by the 2040s compared to > 50% in the 2010s, and approximately 2 °C warmer. Fisheries management reference points (*F*_MSY_ and *B*_MSY_) for demersal fish (cod, haddock) were projected to increase by around 6%, indicating higher productivity. However, planktivorous fish (capelin, herring) reference points were projected to decrease by 15%, and upper trophic levels (birds, mammals) were strongly sensitive to planktivorous fish harvesting. The results indicate difficult trade-offs ahead, between harvesting and conservation of ecosystem structure and function.

**Supplementary Information:**

The online version contains supplementary material available at 10.1007/s13280-021-01616-9.

## Introduction

The effects of global warming are more pronounced in the Arctic than anywhere else on the planet (IPCC 2019). Sea-ice retreat is having profound effects on entire marine food webs in Arctic seas, many of which are already heavily exploited particularly by fishing. Hence these regions represent some of the most urgent cases for adopting an Ecosystem Approach to Fisheries (EAF) (Garcia et al. 2003; Holsman et al. 2020). EAF is defined as “striving to balance societal objectives, by applying an integrated approach to fisheries taking into account the interactions between biotic, abiotic and human components of ecosystems” (FAO 2003).

Fisheries in the Barents Sea are dominated by cod, haddock and capelin (ICES 2019). In the past harvesting of harp seal and whales has been a substantial activity but is now much reduced. Norway and Russia are the main fishing nations, but vessels from EU states, UK, Faroe Islands, Greenland and Iceland also operate in the region. The stand-out feature of the fisheries since the 1960s has been a large surge in capelin abundance in the 1970s (ICES 2019). Catches increased from almost zero in the 1960s to 3 million tonnes (MT), and back down to less than 0.2 MT in the 2010s. Meanwhile, demersal fish catches have fluctuated between 0.5 and 1.5 MT annually. Invertebrate fisheries using trawls and creels have targeted Northern prawn, red crabs, and snow crabs, the latter two being introduced and invasive species, respectively. Discarding is very low since Norway introduced an obligation to land all catches in 2009. Nevertheless, there is evidence of discarding of fish by shrimp trawlers (Breivik et al. 2017). Bycatch in commercial fisheries includes seabirds, seals and cetaceans (mainly porpoises) in gillnet and longlines, with some larger whale entanglement in creel lines. Whale hunting is concentrated on Minke whales and the catch has declined from around 4000 animals per year in the 1950s to around 500 in the 2010s. Seal hunting is presently a subsidised artisanal activity in Norway.

Annual species-by-species assessments for 15 fish and invertebrate stocks in the Barents Sea are carried out by the ICES Arctic Fisheries Working Group (e.g. ICES 2020), and total allowable catches (TACs) were introduced for most stocks in the 1980s. For those species with high quality assessments, fishing mortalities (F) and spawning-stock biomasses (B) are evaluated against their expected values at maximum sustainable yield (MSY). The criteria for being within safe biological limits are *F*_current_ < *F*_MSY_ and *B*_current_ > *B*_MSY_. Cod and haddock have been outside safe limits (*F* > *F*_MSY_) throughout the 1960s–1999s. Since 2009/2010 fishing mortality rates have been reduced to less than F_MSY_ and biomasses have increased dramatically above B_MSY_ (ICES 2019, 2020).

The environmental (bottom-up) causes of fluctuations in capelin, cod and higher trophic level abundances in the Barents Sea, and how these have interacted with fisheries, have been studied by research on ecological processes, statistical analysis of historical data (e.g. Stige et al. 2019; see summary in Appendix S1) and various modelling investigations (Appendix S2). From a modelling perspective, there have been four main types of studies—(i) multi-species models of a restricted subset of mainly fish species (e.g. GADGET; Lindstrøm et al. 2009), (ii) biogeochemical models focussing mainly on the lower trophic levels (e.g. SINMOD; Slagstad and McClimans 2005), (iii) food web models focussing mainly on the upper trophic levels (e.g. Ecopath with Ecosim; Skaret and Pitcher 2016), and (iv) end-to-end models which try to combine the upper and lower trophic levels (e.g. Atlantis; Hansen et al. 2016).

Models to address the issues underpinning an EAF need to represent charismatic megafauna such as marine, and where appropriate, maritime, mammals and seabirds as well as commercially exploited fish stocks, and the linkages to biogeochemical fluxes driven by physics. Achieving this requires strategic simplifications at all levels so that the models are practically usable. Here we use two different types of models—StrathE2EPolar and ECOSMO-Polar which are both based on functional groupings of taxa. The former is a low spatial (vertical and horizontal) resolution exploratory model spanning the end-to-end system from physics to megafauna. The latter is a high vertical resolution physical-biological model of the lower trophic levels. By comparing results from the two models we are able to assess the adequacy with which the low resolution fully end-to-end model represented the all-important primary production process at the base of the food web. We used the models, firstly to simulate the functioning of the present-day (2011–2019) Barents Sea, secondly to project the effects of future (2040–2049) environmental conditions on the ecology, and finally to examine the sensitivity to fishing in each climate period.

## Materials and methods

### StrathE2EPolar model

**Fig. 1:**
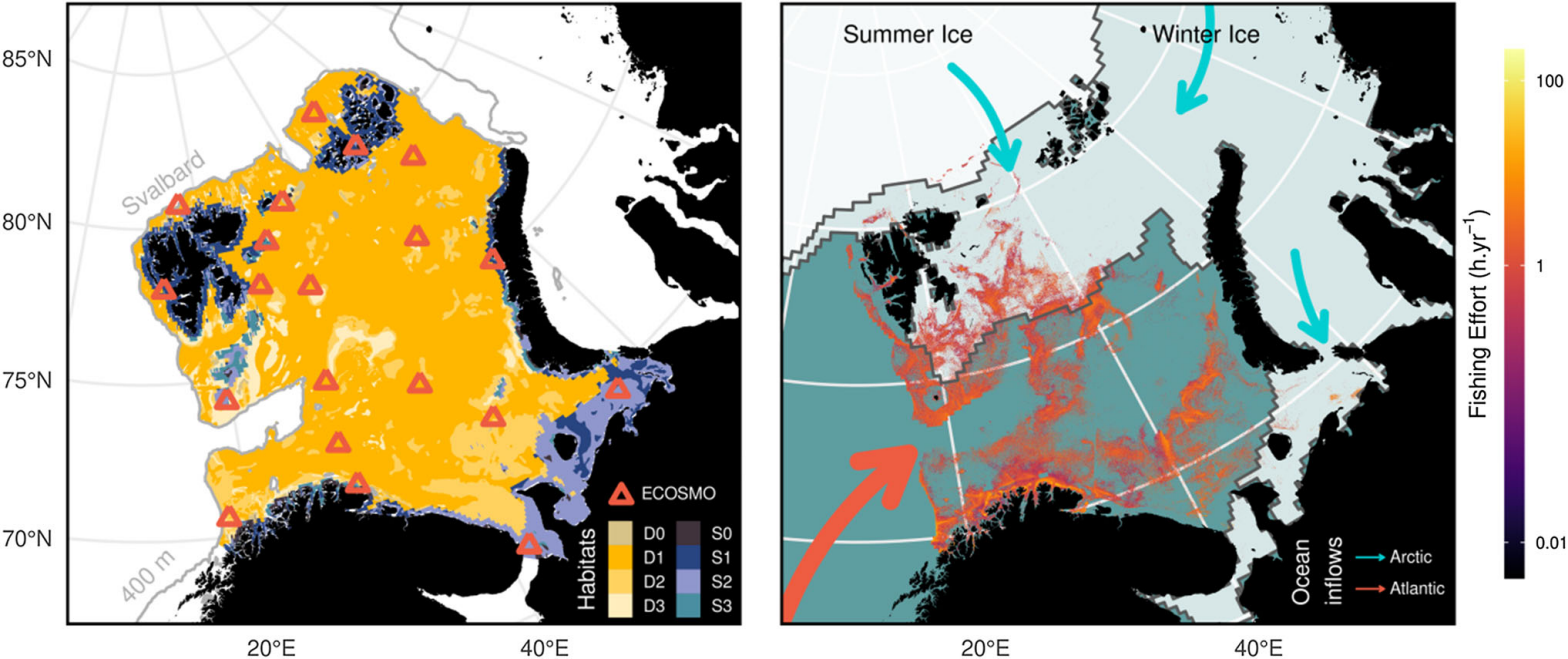
Study area map. The left panel shows the StrathE2EPolar model domain in the Barents Sea. The domain is split horizontally into an inshore zone (blues) and an offshore zone (yellows). The offshore zone water column is divided vertically into upper (surface—60 m) and lower layers representing euphotic and disphotic strata. The seabed in each zone is split into four sediment classes (0–3, Rock, Fine, Medium, Coarse), yielding 8 habitats, based on a synthesis of data from the Geological Survey of Norway (www.ngu.no/en/news/new-seabed-sediment-map-barents-sea). The locations of ECOSMO water columns are indicated by triangles. The right panel provides environmental context; average sea-ice extent in the maximum and minimum months for 2011–2019 derived from ERA5 (https://doi.org/10.24381/cds.f17050d7), water masses flowing into the model domain, and mean annual fishing activity distribution according to Global Fishing Watch for 2012–2016 within the model domain (Kroodsma et al. 2018)

The StrathE2EPolar model builds on an existing temperate shelf-sea fisheries—food web model (StrathE2E2) developed as a package for the R statistical computing environment (www.r-project.org/about.html; https://CRAN.R-project.org/package=StrathE2E2; Heath et al. 2021). StrathE2EPolar is also available as an R-package (https://marineresourcemodelling.gitlab.io/sran/index.html)—version 2.0.0 was used in this study. The chemical and biological groups represented in the model are shown in Table 1. StrathE2E models are spatially resolved into an inshore and offshore zone, the former with a single water layer, the latter with two layers representing the euphotic and disphotic strata. The seabed in each zone is sub-divided into four sediment habitats (Fig. 1). Extensions of StrathE2E to create StrathE2EPolar were the inclusion of nutrient, ice algae and detritus dynamics in sea-ice, the effects of ice and snow on light penetration into the sea, and their effects on the feeding ecology, mortality and active migration rates of higher trophic levels. Documentation on the formulation of these extensions is also available at the package gitlab site. The model outputs data at daily intervals.

**Table 1:** Ecological guilds or classes of dead and living material included in the StrathE2EPolar and ECOSMO-Polar models. Terms marked * were added to the respective source models in order to create the polar versions. Detritus and bacteria were represented as a composite guild in both models

Type of guild or class	StrathE2EPolar	ECOSMO-Polar
Dissolved inorganic nutrients	• Nitrate in snow*, ice*, water column, sediment porewaters • Ammonia in snow*, ice*, water column, sediment porewaters	• Nitrate in ice*, water column, sediment porewaters • Ammonia in ice*, water column, sediment porewaters • Phosphate in ice*, water column, sediment porewaters • Silicate in ice*, water column, sediment porewaters
Dead organic material and bacteria	• Suspended detritus and bacteria • Ice detritus and bacteria* • Labile sediment detritus and bacteria • Refractory sediment detritus • Macrophyte debris • Corpses • Fishery discards	• Suspended detritus and bacteria • Dissolved organic material and bacteria • Ice detritus and bacteria* • Labile sediment detritus and bacteria
Primary producers	• Phytoplankton • Ice algae* • Macrophytes	• Flagellates • Diatoms • Ice algae*
Zooplankton	• Omnivorous zooplankton • Carnivorous zooplankton • Larvae of planktivorous fish • Larvae of demersal fish • Larvae of suspension and deposit feeding benthos • Larvae of carnivore and scavenge feeding benthos	• Microzooplankton • Mesozooplankton
Benthos	• Suspension and deposit feeders • Carnivore and scavenge feeders	• Macrobenthos
Fish	• Planktivorous • Migratory • Demersal (benthic-piscivorous)	
Upper trophic levels	• Seabirds • Pinnipeds • Cetaceans • Maritime mammals (polar bears)*

### ECOSMO-Polar model

The ECOSMO-Polar model builds on the existing ECOSMO-E2E model for the North and Baltic Seas (Daewel et al. 2019). The extensions of ECOSMO-E2E to create the ECOSMO-Polar was the implementation of a sympagic (sea-ice biogeochemistry) module, including ice algae, detritus and 4 nutrients groups (nitrate, ammonia, phosphate and silicate), and the sympagic interaction with the existing pelagic and benthic systems (Benkort et al. 2020; Table 1). In addition, state variables for chlorophyll were included to take account of the variable chlorophyll content in phytoplankton (Yumruktepe et al. unpubl.). ECOSMO-Polar was run in a 1-D vertical mode in this study with output at 30 min intervals (subsequently aggregated to daily averages), as in Benkort et al. (2020).

### Input data for the models

**Fig. 2:**
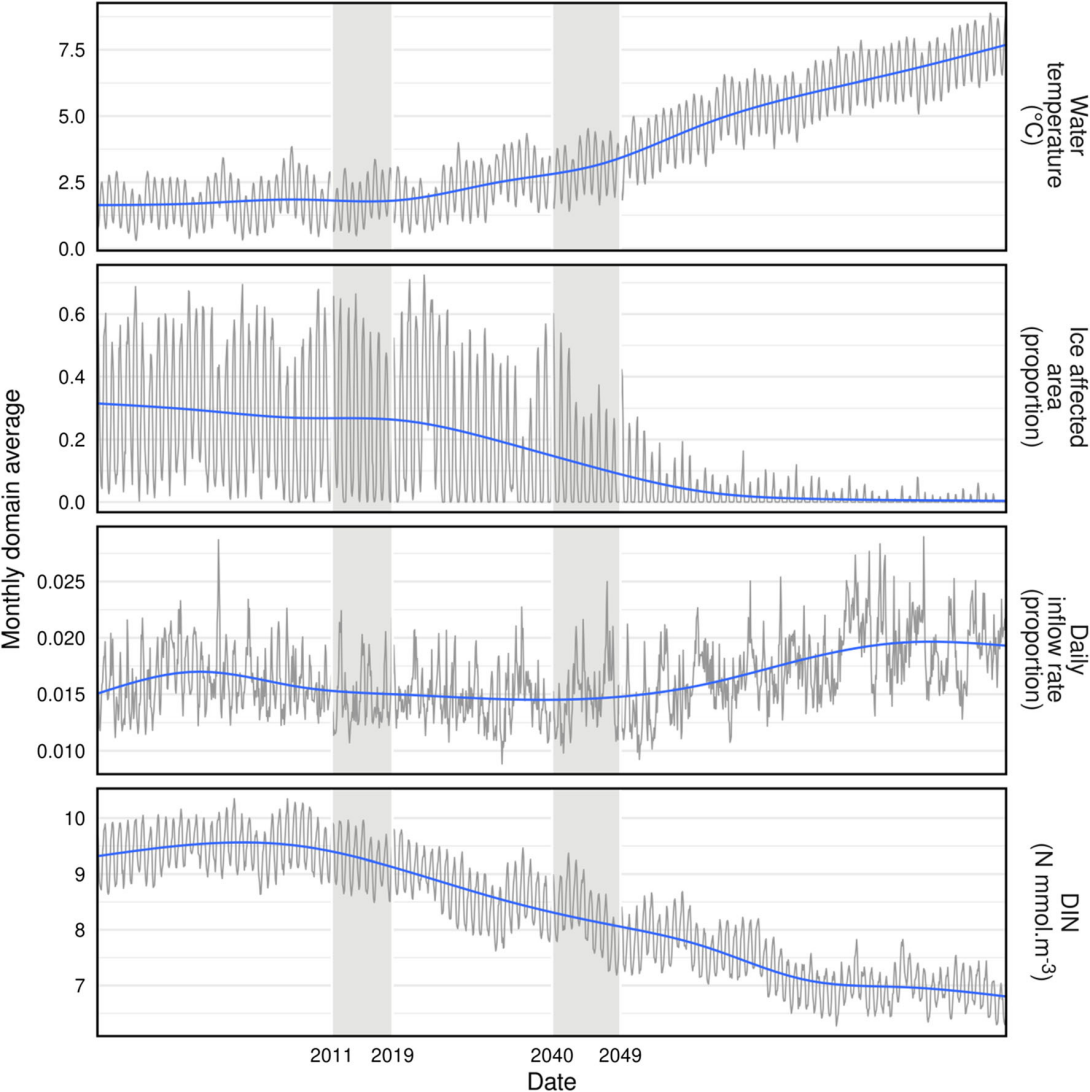
Time series of data from NEMO-MEDUSA RCP8.5 model outputs, 1980–2100. In each panel the grey line represents monthly values and the blue line a smoothed trend. Monthly values are the means of all pixels falling within the 3-D volume of the StrathE2EPolar model domain by month, between 1st January 1980 and 31st December 2099. Vertical grey bars indicate the time periods contributing driving data to this study. Ice affected area is the proportion of sea surface area with an ice cover of ≥ 15% (ice cover being the proportion of a pixel in the model output which is covered by ice). Inflow rate is the daily average volume of water flowing into the model region as a proportion of domain volume. DIN corresponds to dissolved inorganic nitrogen concentration (nitrate plus nitrite and ammonia) in millimolar units

StrathE2EPolar was driven by time varying physical and chemical data extracted from output of the 3-dimensional, quarter-degree latitude x longitude NEMO-MEDUSA earth system physical-biogeochemical model (Yool et al. 2015). NEMO-MEDUSA was run from 1980 to 2100 with atmospheric forcing assuming IPCC representative concentration pathway emissions scenario “RCP8.5” (Riahi et al. 2011). The driving data for StrathE2EPolar were assembled for the periods 2011–2019 (referred to here as the “2010s”; representing present-day conditions) and 2040–2049 (the “2040s”). The 2040s period was chosen to represent the future state as this is the onset of nearly year-round ice-free conditions in the Barents Sea, according to NEMO-MEDUSA (Fig. 2). Two types of data were extracted from NEMO-MEDUSA for input to StrathE2EPolar: (1) climatological (2010s and 2040s) annual cycles of monthly area or volume averaged values over each StrathE2EPolar water column zone and layer, and (2) climatological annual cycles of monthly averaged or integrated data at the external boundaries of the StrathE2EPolar domain. Data of the first type were water temperature, vertical diffusivity at the interface between vertical layers in the offshore zone, ice (and snow) extent, cover and thickness, and daily integrated incident irradiance. Data of the second type were daily integrated ocean and river water inflow volumes across the external boundaries, and the boundary concentrations of dissolved inorganic nitrogen (DIN), detritus and phytoplankton.

Other StrathE2EPolar driving data were significant wave height in the inshore zone (CERA-20C ‘Ocean Wave Synoptic Monthly Means’ product accessed through ECMWF); monthly averaged annual cycles of wet and dry atmospheric nutrient deposition rates assembled from the EMEP data centre (https://www.emep.int/mscw/mscw_moddata.html); riverine nutrient concentrations from Holmes et al. (2021), and suspended particulate matter (SPM) in the inshore zone and upper layer of the offshore zone from remote sensing data (Globcolour L3b; ftp://ftp.hermes.acri.fr/GLOB/merged/month/). Apart from wave height, we were unable to identify sources of future projections for these inputs, so we assumed that they will remain constant into the 2040s. Further details are provided in Appendix S3.

Atmospheric driving data for ECOSMO-Polar simulations were prescribed from the MERRA2 reanalysis (Gelaro et al. 2017), which is available with a 50 km horizontal resolution and hourly instantaneous output for the “2010s”. Atmospheric variables from HadGEM2-ES RCP8.5 (Jones et al. 2011; data accessed through www.isimip.org) were used for the “2040s”, consistent with driving conditions of NEMO-MEDUSA. The relevant forcing variables were air temperature, pressure and humidity, wind velocities and shortwave radiation.

Data to configure the fishing fleet model component of StrathE2EPolar were assembled for the period 2011–2019 from the Norwegian Directorate of Fisheries, EU STECF (https://stecf.jrc.ec.europa.eu/dd/fdi/spatial-land-map), Global Fishing Watch (Kroodsma et al. 2018), and regionally integrated landings by nation and species from the ICES/FAO data centre (FAO areas 27.1 and 27.2.b). Additional data on artisanal seal harvest, discards of fish by shrimp trawlers, catches by the recreational/tourism/subsistence fishers in Norway and Russia, and by-catches of seabirds, seals and cetaceans in coastal gillnet and longline fisheries, were assembled separately from a range of literature and data sources. Full documentation of the workflow to generate these input data to the fleet model is available separately (https://marineresourcemodelling.gitlab.io/sran/index.html), and a summary of the fishing gears represented in the model in Appendix S4.

### Data for model optimization and validation

Observational data on the state of the Barents Sea ecosystem during the 2010s modelling period was assembled from literature sources. These data formed the target for a computational optimization of the StrathE2EPolar parameters by simulated annealing (Kirkpatrick et al. 1983; Heath et al. 2021). The data and their sources are listed in Appendix S5. The outcome of the optimization was a parameter set which produced the best fit of the model to the observations (Fig. S3).

Independent validation of models was carried out by comparison with satellite data on chlorophyll concentrations, and acoustic survey data on fish and zooplankton distributions. Ocean colour sensing data, calibrated as chlorophyll concentrations, were downloaded at 1 km resolution for 2011–2019 (https://resources.marine.copernicus.eu). The 25th, 50th, and 75th percentiles of monthly aggregated pixel chlorophyll concentrations were calculated for the inshore and offshore zones of StrathE2EPolar (Fig. 1).

Raw Simrad EK60 echosounder data (18, 38 and 120 kHz; calibrated as per Demer et al. 2015) collected annually in August and September between 2011 and 2016 as part of the Barents Sea Ecosystem Survey (Fig. S4; Eriksen et al. 2018) were obtained from the Norwegian Marine Data Centre. Details of the processing to generate Nautical-Area Scattering Coefficients (NASC, m^2^ nmi^−2^: average received echo energy over a given depth range scaled up to a square nautical mile) are provided as Appendix S6. Mean NASC values for both fish and macro-zooplankton were computed for both the inshore and offshore zones of the StrathE2EPolar model domain. Bootstrap sampling was used to calculate confidence intervals. NASC values were also binned into a 0.5 by 0.5 degree grid and averaged to map the spatial distribution of fish and macro-zooplankton.

### Strategy for using the models

In all cases the models were run to a steady state (until they produced repeating annual cycles of outputs with repeating annual cycles of input driving data; 60–100 years for StrathE2EPolar depending on scenario conditions). Under these conditions the results from the models were completely independent of their initial conditions. The models were used to conduct 3 types of experiments as described below.

Experiment 1: Consistency between models and independent observational data. StrathE2EPolar has a relatively parsimonious representation of physics and biogeochemistry. We assessed its effectiveness at simulating the base of the food web by comparison with ECOSMO-Polar which has a more elaborate representation of physics, microbes and autotrophs in terms of vertical resolution, nutrient and guild diversity.

ECOSMO-Polar was run at 20 locations covering the inshore and offshore zones of the StrathE2EPolar model with 2010s driving data. Within each zone, between-site variations in phytoplankton chlorophyll concentrations were averaged over the upper 60 m and summarised by the mean, median and quantiles of the results across all sites within each zone. These distributional properties were then compared with credible intervals of equivalent outputs for each zone from StrathE2EPolar generated by a likelihood-weighted Monte Carlo analysis of parameter uncertainty (full details of the methodology available from the R-package gitlab site), and with mean, median and quantiles of the independent observational data for the 2010s period which were not used in the optimization processes for either model (annual cycles of satellite remote sensing data on chlorophyll).

In addition, August and September data on inshore and offshore macro-zooplankton and fish biomass from StrathE2EPolar (these variables were not available from ECOSMO-Polar) were compared with August/September acoustic survey data. Macro-zooplankton biomass from StrathE2EPolar was the sum of the carnivorous zooplankton and fish larvae guilds; fish biomass was the sum of planktivorous, migratory and demersal fish guilds.

Experiment 2: Projection of future ecosystem state in the 2040s. Both StrathE2EPolar and the 20 ECOSMO-Polar site models were run to a steady state with 2040s external driving data from NEMO-MEDUSA. For StrathE2EPolar, inputs to the 2040’s fishing fleet model were assumed to be identical to the 2010s. Annual mean masses of each of the state variables in each model were then expressed as a percentage change relative to the corresponding properties of the 2010s model. ECOSMO-Polar state variable outputs were aggregated across functional guilds so as to correspond with the coarser guild resolution of StrathE2EPolar.

Experiment 3: Ecosystem sensitivity to fishing. For StrathE2EPolar only (ECOSMO-Polar did not include any representation of fish or fishing), the 2010s and 2040s models were run to a steady state for each member of a sequence of increasing values of harvest rate on planktivorous and demersal fish. Here, harvest rate was the daily fish mortality rate due to fishing—related to the proportion of fish biomass captured per day. For both periods, the rates were expressed as multiples of 2010s mean values as determined from the observational data. Proportions of total effort attributable to each gear type and their spatial distributions (inshore-offshore), discard rates and seabed abrasion, were held constant across all runs. For each decadal period, two sets of runs were carried out: (a) increments of fishing mortality on planktivorous fish with demersal fishing mortality held constant at the 2010s value, and (b) increments of fishing mortality on demersal fish with planktivorous fishing mortality held constant at the 2010s value.

Simulated catches in each of the fishing runs mapped out the standard dome-shaped “yield curves'' for planktivorous and demersal fish (fishing mortality rate vs catch) and the corresponding fishing mortality rate vs biomass curves, which form the basis for setting fisheries management plan reference points, i.e. the biomass and fishing mortality rate at maximum sustainable yield (*B*_MSY_ and *F*_MSY_). The present status of a fishery is often expressed by the ratios *B*_current_/*B*_MSY_ and *F*_current_/*F*_MSY_. The credibility of the 2010s fishing sensitivity results was assessed by comparing these indicators derived from the simulations with the guild-level value aggregated from individual species stock assessments produced by the ICES Arctic Fisheries Working Group (ICES 2020).

In addition to yield curve outputs, the results were used to assess the sensitivity of annual average fish, seabird, pinniped, cetacean and maritime mammal (polar bear) biomasses to each fishing scenario.

## Results

### Experiment 1

**Fig. 3:**
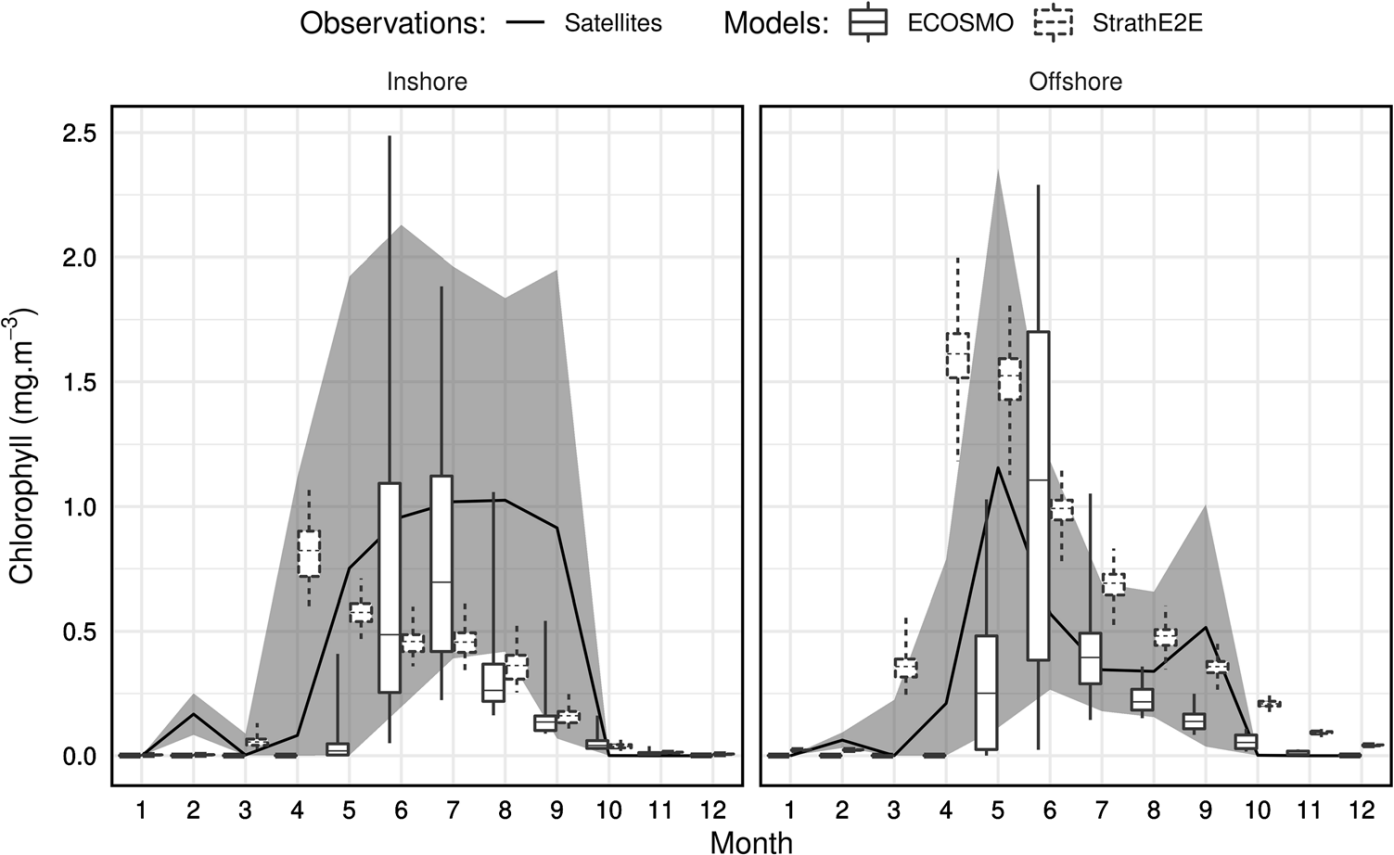
Phytoplankton chlorophyll comparison between model outputs and observations for the climatology of the 2010s. Box plots show the median and interquartile range, with whiskers indicating 0.5th and 99.5th percentiles. The shaded area indicates the interquartile range for satellite observations (https://resources.marine.copernicus.eu/?option=com_csw&view=details&product_id=OCEANCOLOUR_ARC_CHL_L4_REP_OBSERVATIONS_009_088), with the median as a solid line. The range bars for ECOSMO output represent spatial variability between model sites within each zone. For StrathE2EPolar the range bars represent credible intervals of model output due to parameter uncertainty

The 2010s annual cycles of inshore and offshore phytoplankton chlorophyll concentrations from StrathE2EPolar and ECOSMO-Polar were consistent with each other, especially in the offshore zone (Fig. 3). An intense spring bloom in April/May was followed by declining concentrations through the summer and autumn. In both models the concentrations were lower in the inshore zone. These inshore and offshore patterns were replicated in the satellite data, though the absolute levels were different. Satellite-derived concentrations were consistent with the models in the offshore zone but higher in the inshore, although with high year-to-year variability. Inshore waters off northern Norway are known to contain high concentrations of Coloured Dissolved Organic Matter of terrestrial origin (Nima et al. 2016) which is interpreted as chlorophyll in satellite reflectance data. Although the Copernicus data have been corrected for so-called Type II waters to account for this effect, the generic algorithms cannot accurately account for all situations.

**Fig. 4:**
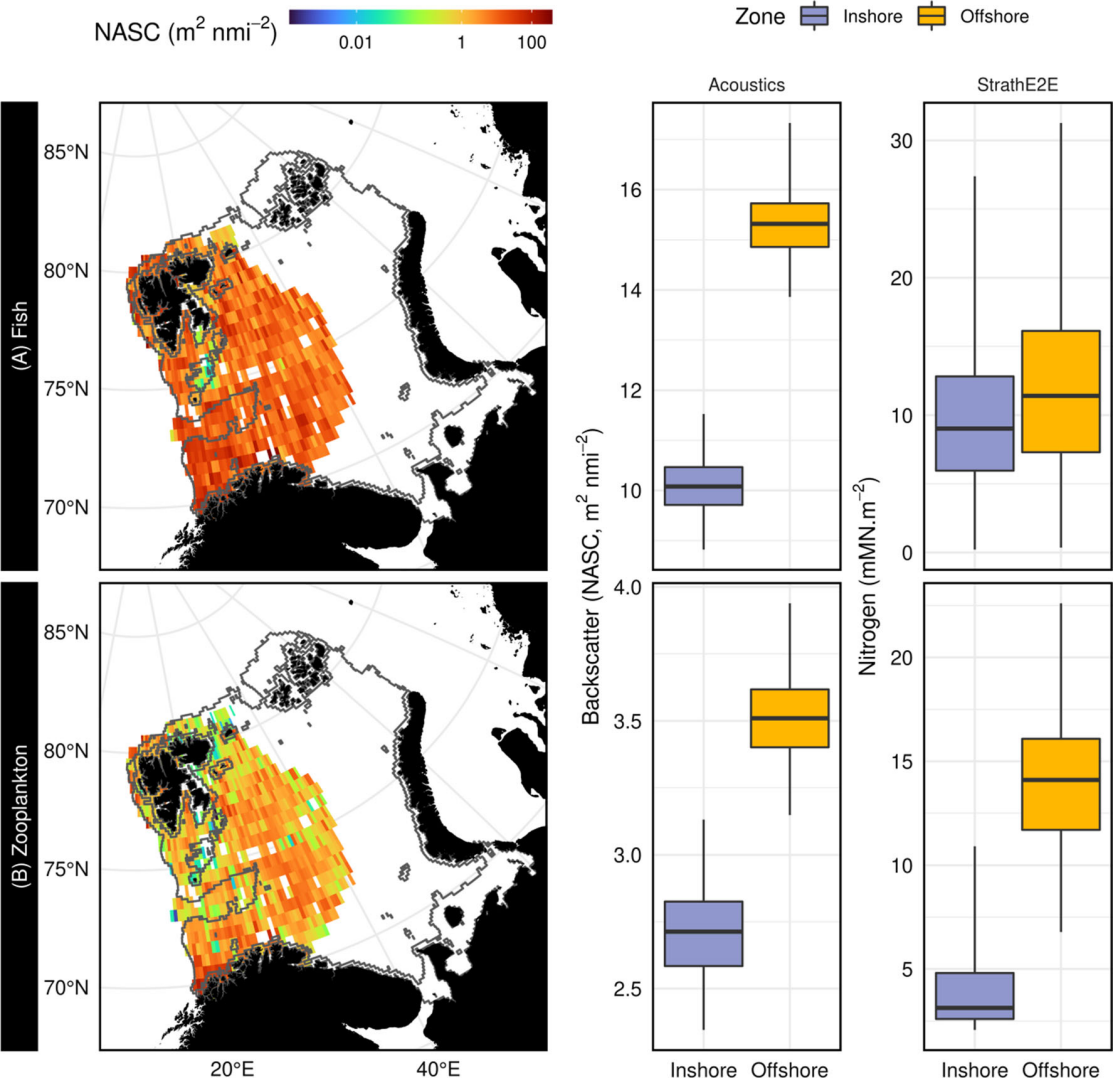
StrathE2EPolar model predictions for inshore and offshore zone compared with echo sounder observations. Upper row: fish biomass, lower row macro-zooplankton. Maps to the left show depth integrated acoustic backscattering intensity (NASC) binned into a 0.5 by 0.5 degree regular grid and averaged over August and September 2011–2016. Centre column: interquartile ranges (0.5th, 25th, median, 75th and 99.5th centiles) of NASC area-density values over the inshore and offshore zones of the model domain. Right column: Credible interquartile ranges of August and September 2010s mean inshore and offshore zone macro-zooplankton (carnivorous zooplankton and fish larvae guilds combined) and fish (planktivorous, migratory and demersal guilds combined) area densities from StrathE2EPolar model, generated by Monte Carlo simulations

August and September output from the 2010s StrathE2EPolar model and the echosounder observations both showed higher depth integrated concentrations (area densities) of macro-zooplankton and fish offshore than inshore (Fig. 4). Lower values of fish and macro-zooplankton acoustic backscattering intensity occurred in the shallower regions (i.e. inshore zone) especially east of the Svalbard archipelago in the north-west of the region (Fig. 4). This is consistent with relatively low primary production associated with cold Arctic water currents that flow into the Barents Sea from the north and north east (Eriksen et al. 2018).

### Experiment 2

**Fig. 5:**
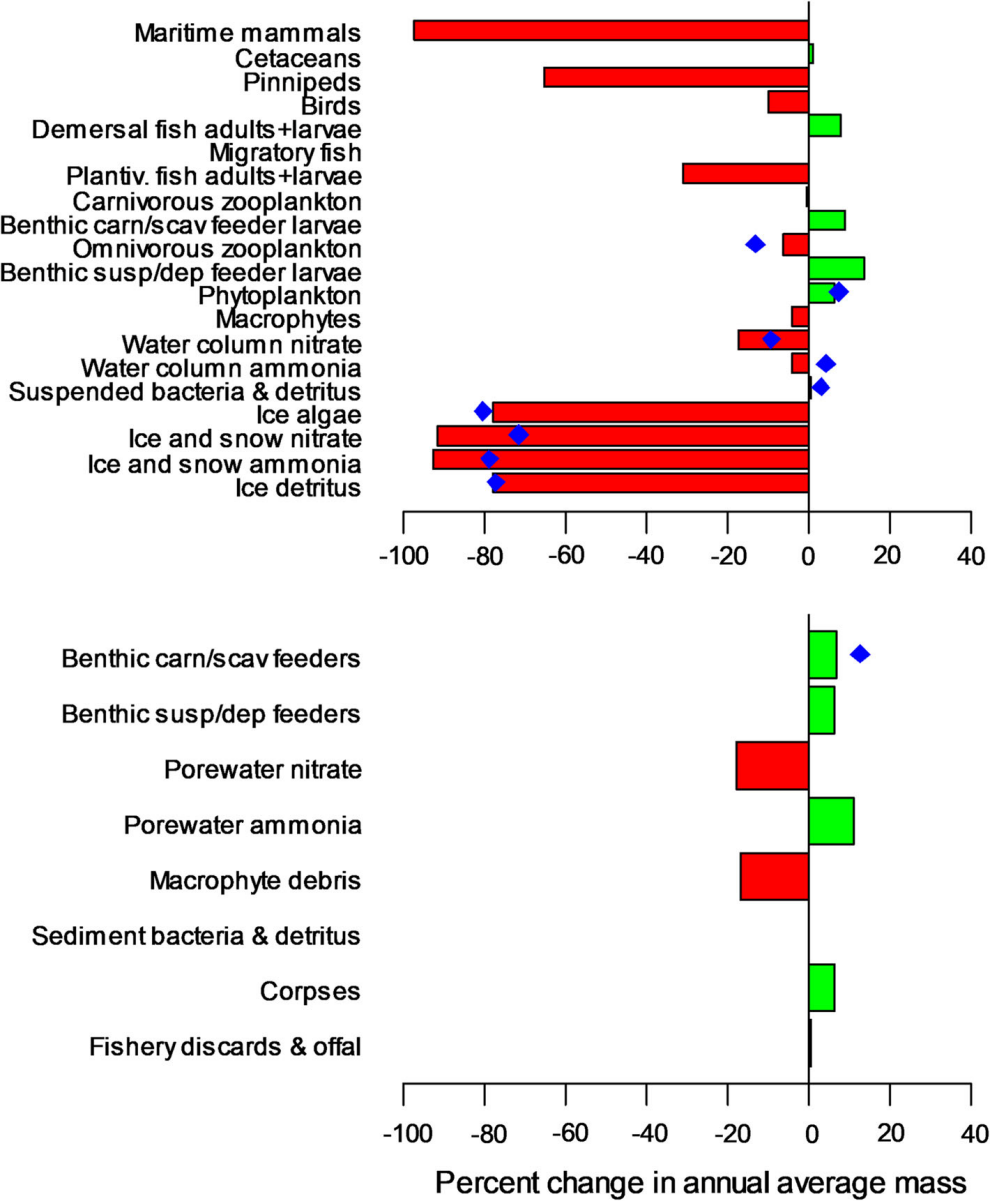
Differences in model annual average masses of food web components between the 2040s and 2010s. Upper panel: Water column and ice properties, lower panel seabed properties. Red and green refer to StrathE2EPolar results. Blue symbols refer to ECOSMO-Polar (which has a more restricted food web). Green bars, and symbols to the right, indicate that the variable was larger in the 2040s than in the 2010s, and vice versa for red bars and symbols to the left. Annual net primary production (phytoplankton and ice algae combined) derived by the StrathE2EPolar model was 844.5 mMN m^−2^ year^−1^ in the 2010s and 912.3 mMN m^−2^ year^−1^ in the 2040s (equivalent to 67.1 and 72.5 gC m^−2^ year, respectively assuming Redfield equivalence)

Comparison of 2010s and 2040s annual mean masses of the state variables in StrathE2EPolar (aggregated to the whole model domain; Fig. 5) showed the combined effects of bottom-up and top-down cascading effects in the food web. Modelled annual net primary production (phytoplankton and ice algae combined, but > 99% due to phytoplankton) increased by 8% between the 2010s and 2040s, driven to the loss of ice cover and consequent increased sub-surface light intensity (844.5 mMN m^−2^ year^−1^ in the 2010s; 912.3 mMN m^−2^ year^−1^ in the 2040s, equivalent to 67.1 and 72.5 gC m^−2^ year^−1^ respectively assuming Redfield equivalence). This was reflected in a similar percentage increase in annual average phytoplankton biomass, but did not uniformly cascade up the food web to mid-trophic levels (zooplankton and fish). Cetaceans, birds and migratory fish showed only small changes in biomass between the 2010s and 2040s model runs since in each of these cases their migration patterns took them outside of the Barents Sea model domain for part of the annual cycle during which their dynamics were un-modelled. Hence these guilds were to some extent buffered against changes in food web productivity within the domain. Benthic guilds were positively affected by the loss of ice and warming in the 2040s in StrathE2EPolar due to the increased flux of detritus to the seabed. Water column nitrate concentrations were lower in the 2040s due to two factors (a) increased uptake by phytoplankton, and (b) reduced external influx across the model open boundary due to changing transport fluxes and dissolved inorganic nitrogen (DIN) concentrations in NEMO-MEDUSA (Fig. 2; annual integrated DIN influx to the model domain: 2010s, 8870 mMN m^−2^ year^−1^; 2040s, 7855 mMN m^−2^ year^−1^). Ice and snow nutrient masses showed large decreases due to the loss of ice extent and thickness. StrathE2EPolar and ECOSMO-Polar were in good agreement as to the direction and extent of simulated changes for their overlapping guilds.

### Experiment 3

**Fig. 6:**
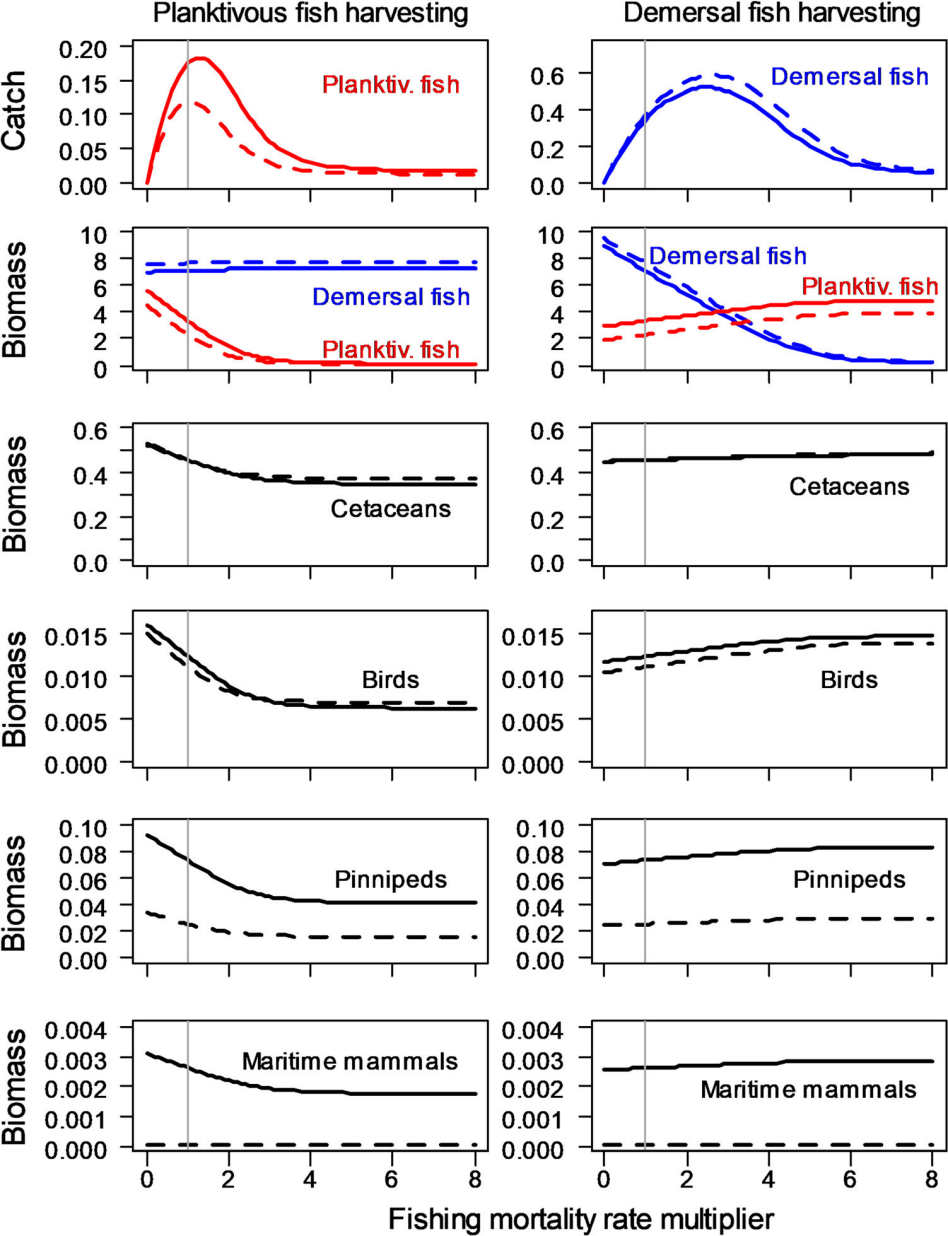
StrathE2EPolar 2010s and 2040s sensitivity to fishing mortality. Solid lines 2010s, dashed lines 2040s. Units for catch are mMN m^−2^ year^−1^. Units for biomass are mMN m^−2^. X-axis of each panel shows multiples of the 2010s fishing mortality rate for either plantivorous or demersal fish. Hence the vertical grey line at *x* = 1 indicates the rate effective in the 2010s. Left column shows the effects of varying planktivorous fishing mortality whilst keeping demersal fishing constant. Vice-versa for the right column—varying demersal fishing whilst keeping planktivorous constant

The 2010s fishery yield curve for demersal fish (Fig. 6) suggested that *F*_2010s_/*F*_MSY_ during this period was 0.41 (i.e. *F*_MSY_ = 2.47 × *F*_2010s_), which was reasonably consistent with the combined ICES stock assessment outputs for Arctic cod, haddock and saithe (Table 2). No stock assessment estimates of *F*_2010s_/*F*_MSY_ were available for planktivorous species, but StrathE2EPolar indicated that the ratio was around 0.78. The 2010s values of *B*_2010s_/*B*_MSY_ from the model for planktivorous and demersal fish were 1.2 and 1.6 respectively, indicating that 2010s biomass was higher than that obtained if the guilds had been fished at their respective MSY rates. ICES Arctic Fisheries WG estimates of the ratios were also > 1, but larger than from our model (ICES 2019; 4.3 and 4.9, respectively). However, The ICES values of *B*_current_/*B*_MSY_ have been highly variable over the 2011–2019 period and the latest values are around 2.1 and 3.0, respectively. Nevertheless, the general evidence from the stock assessments that the guilds are being exploited conservatively at fishing mortalities less than F_MSY_ is clearly replicated by the 2010s model.

**Table 2:** Fisheries metrics for planktivorous and demersal fish in the 2010s and 2040s extracted from the results of Experiment 3 using the StrathE2EPolar model (Fig. 6), and comparable measures from national catch statistics, ICES stock assessments and the Barents Sea Ecosystem Surveys. Upper half of the table shows catch and fishing mortality (*F*) data, lower half shows biomass (*B*) data. Catch and biomass conversions between model millimolar nitrogen units (mMN m^−2^ year^−1^ and mMN m^−2^) and thousands of tonnes live weight, assuming nitrogen contents of 2.038 and 1.340 mMN g WW^−1^ for planktivorous and demersal fish respectively, and a surface area for the Barents Sea model domain of 1.60898 × 10^6^ km^2^. National statistics on catch data for the 2010s were assembled from the Norwegian Directorate of Fisheries, EU STECF, and the ICES/FAO landings data for areas 27.1 and 27.2.b (see text for details). Data on *F*_2010s_/*F*_MSY_ for cod, haddock and saithe in the 2010s were digitised from the 2020 ICES Arctic Fisheries Working Group Report (ICES 2020, p. 27) and scaled to the whole demersal fish guild using trawl survey species composition data from the annual Norwegian/Russian Barents Sea Ecosystem Survey (BSES; Protozorkevich et al. 2020). Data on *B*_at F2010s_/*B*_MSY_ for planktivorous fish (capelin and beaked redfish) and demersal fish (cod and haddock) were digitised from ICES (2019; Fig. 10)

Source	Metric	Units	Planktivorous fish		Demersal fish	
			2010s	2040s	2010s	2040s
Model	Catch at *F*_2010s_	mMN m^−2^ year^−1^	0.175	0.118	0.341	0.367
Model	Catch at *F*_2010s_	× 10^3^ tonnes year^−1^	137.8	93.4	409.1	440.6
National stats	Catch	× 10^3^ tonnes year^−1^	142.6		401.1	
Model	MSY	mMN m^−2^ year^−1^	0.182	0.119	0.523	0.589
Model	MSY	× 10^3^ tonnes year^−1^	143.8	93.6	627.8	706.7
Model	*F*_2010s_/*F*_MSY_		0.781	0.943	0.405	0.383
ICES/BSES	*F*_2010s_/*F*_MSY_				0.572	
Model	Biomass at *F*_2010s_	mMN m^−2^	3.343	2.299	7.136	7.673
Model	Biomass at *F*_2010s_	× 10^3^ tonnes	2636.5	1813.7	8563.5	9207.7
BSES	Biomass	× 10^3^ tonnes	3020.1		3747.4	
Model	*B*_MSY_	mMN m^−2^	2.744	2.181	4.475	4.756
Model	*B*_MSY_	× 10^3^ tonnes	2164.2	1720.3	5369.6	5707.0
Model	*B*_at F2010s_/*B*_MSY_		1.219	1.054	1.595	1.613
ICES	*B*_at F2010s_/*B*_MSY_		4.324		4.909	

Modelled demersal fish biomass was only weakly (positively) sensitive to planktivorous fishing in the 2010s, though the diet composition became more benthivorous as planktivorous fish were depleted by harvesting. The positive sensitivity was partly because planktivorous fish were predators on demersal fish larvae, and partly an indirect effect through predation on carnivorous zooplankton (which were a component of the diet of demersal fish). In contrast, the biomasses of all the higher trophic level guilds (birds, pinnipeds, cetaceans and maritime mammals) were directly related to planktivorous fish biomass (inversely related to planktivorous fishing mortality). In the case of birds, pinnipeds and cetaceans, examination of the fluxes between guilds showed that this was due to a direct diet dependency on planktivorous fish. In the case of maritime mammals, it was due to an indirect effect through dependence on pinnipeds.

The 2040s simulations showed that with the exception of pinnipeds and maritime mammals the simulated effects of warming and ice loss on guild biomasses were substantially smaller than the effects of fishing, especially that on planktivorous fish (Fig. 6). MSY for demersal fish was projected to increase to 112% of the 2010s value (with a corresponding increase in *F*_MSY_), whilst planktivorous fish MSY was projected to decrease to 65% of the 2010s value. (Fig. 6, Table 2). These results were in keeping with those from Experiment 2, and show that the productivity of the demersal fish guild is projected to increases by the 2040s, whilst that of the planktivorous fish is projected to decrease.

## Discussion

### Model strengths, assumptions and uncertainties

StrathE2EPolar was conceived as an educational, rapid exploratory, whole-ecosystem-scale tool which can reveal the macroscopic responses to be expected from environmental changes or interventions such as fishing or nutrient emissions. Critically, the model is fast running (< 2 s per simulation year) enabling the hundreds of thousands of annual iterations required for formal parameter optimization, global parameter sensitivity analysis, and computation of credible intervals of model outputs without relying on high performance computing facilities. ECOSMO-Polar is also a versatile, modular code which can be deployed either in high resolution 1-D vertical mode for rapid simulation (as in this study), or in a full 3-D configuration. ECOSMO-Polar and StrathE2EPolar share a common formulation of sympagic biogeochemistry and its coupling to the pelagic system (Benkort et al. 2020). StrathE2Polar includes novel representations of a range of additional sea-ice processes which are absent in other food web models, including ice-dependent migration and feeding efficiency of the high trophic level guilds (birds, pinnipeds, cetaceans and maritime mammals). These features are fundamental to modelling the ecology of the changing Arctic.

A key structural design feature of StrathE2EPolar to achieve the required fast run-times was the coarse spatial compartmentalisation. This is problematic for representation of biogeochemistry due to strong gradients in process rates particularly in the vertical dimension. Hence the need for our comparisons between StrathE2EPolar, ECOSMO-Polar, and independent data not used in the model parameter optimization processes (Experiment 1). The comparison showed that when ECOSMO-Polar results are aggregated up to the spatial and guild granularity of StrathE2EPolar, the two models perform more or less equally well at explaining the annual cycle of phytoplankton chlorophyll derived from remote sensing data. StrathE2EPolar also reproduced the coarse spatial distributions of macro-zooplankton and fish derived from echosounder surveys. Together these results raise our confidence that we can use StrathE2EPolar to draw meaningful conclusions on the whole food web. Other models such as Ecopath with Esosim (EwE) also have low or no spatial resolution, but do not model the biogeochemistry of the system, instead treating primary production as a data-driven boundary condition.

Process parameters in StrathE2EPolar (e.g. maximum uptake rates) are constrained by extensive observational data on the state of the ecosystem in a given time period, to which the model is computationally optimised. However, the optimisation is conditional on the prescribed external driving data. These are the time-varying inputs on physical and chemical boundary conditions (temperature, ice extent and cover, external nutrient fluxes etc.) which were extracted from the NEMO-MEDUSA earth system model and other sources as described in the Methods section. Similarly, ECOSMO-Polar results are conditional on atmospheric driving data from the MERRA2 reanalysis. Hence, all of our results and conclusions are subject to the realism of these inputs. The NEMO-MEDUSA data were from a well-documented run configured to represent the IPCC RCP8.5 high emissions scenario (Yool et al. 2015). In an ideal world, we would test the sensitivity of our ecological models to inputs for different RCP scenarios (Moss et al. 2010) and from different earth system models. However this is a massive task and beyond our means in this project.

### Effects of climate change in the ecosystem

NEMO-MEDUSA projected declining inorganic nutrient concentrations at our model boundaries between the 2010s and 2040s (see also Yool et al. 2015), and a 10% reduction in the annual flux of nutrient into the model domain. Nevertheless, the effects of this declining nutrient flux were outweighed by increased light penetration into the water due to ice loss leading to an 8% increase in net annual primary production in the StrathE2EPolar runs (Experiment 2; Fig. 5). In a temperate shelf-sea situation such an increase in primary production would be expected to propagate more or less uniformly up the food web (Heath et al. 2014). However, in our Barents Sea model this bottom-up effect was more complex due to the various effects of ice loss on high trophic levels. In the real world, foraging birds and cetaceans are constrained by ice because they risk becoming trapped beneath it. The major pinniped species (e.g. harp seal) are ice-edge dependent, since they need to haul out to rest and breed. Bearded seals maintain ice holes enabling them to forage beneath ice cover. Polar bears need ice of sufficient thickness to hunt their preferred prey (pinnipeds). In the absence of ice, they are forced to adopt a land-based existence foraging in the inshore zone. Empirical evidence suggests an increased reliance of land-based polar bears on carrion, birds and especially whale strandings (Laidre et al. 2018). All these processes have been carefully represented in StrathE2EPolar. The most prominent result of this was a strong negative response of pinniped and maritime mammal biomass to the change in environmental conditions between 2010 and 2040s, with predation consequences cascading down the food web. Diagnosing the causes and effects of mid-trophic level responses in the complex food web was difficult given the “collision” between bottom-up and top-down feeding and predation pressures, but the outcome was a reduction in planktivorous fish biomass and increases in demersal fish and benthos.

### Interactions between climate and fishing

The fishing sensitivity simulations (Experiment 3; Fig. 6) have two clear messages for fisheries management: (1) biomasses of fish and higher tropic levels (except pinnipeds and maritime mammals) are more sensitive to fishing than to the environmental changes expected by the 2040s. Hence fisheries management has a key role to play in alleviating the ecosystem consequences of climate warming. (2) The future environment is likely to result in increased harvesting opportunities for demersal fish, with scope for increasing fishing mortality reference points (*F*_MSY_) by about 6%. In fact, increasing demersal fish harvesting could have some ecosystem benefits by reducing predation pressure on planktivorous fish, upon which much of the iconic higher trophic levels depend either directly or indirectly. However, planktivorous fish are likely to come under increasing predation pressure especially from demersal fish, birds and cetaceans, resulting in around 15% reduction in their *F*_MSY_ reference point, leaving no scope for increasing harvesting rates. The best chance of alleviating the climate pressure on pinniped and maritime mammals through an EAF is to restrict planktivorous harvesting. Nevertheless, other additional measures to protect maritime mammals will be required to make a meaningful impact on the projected scale of reduction in their biomass.

### Increased understanding of the Barents Sea ecosystem

StrathE2EPolar and ECOSMO-Polar part of a growing suite of models that have been deployed in the Barents Sea (see Appendix S2). Two key conclusions emerge from our study and other recent models. First, like Sivel et al. (2021) we find that top-down predation pressure is a fundamental feature of the Barents Sea food web (Experiment 3). The surge in planktivorous fish abundance, especially capelin, in the 1970s (Johannesen et al. 2012) may have had some environmental origins but the models indicate a primary cause being relaxation of predation pressure as a result of demersal fish depletion through over-fishing. The process has been reversible—reductions in demersal fishing mortality rates since 2000 have led to a large increase in demersal fish biomass and suppression of planktivorous fish. Second, the direct effects of warming on the physiology of fauna and flora in the food web seem to be less significant than the indirect effects arising from loss of sea-ice. StrathE2EPolar includes explicit representations of ice and ice-dependency. This is an advance on other food web models of the region, e.g. Atlantis (Hansen et al. 2016) which does not include ice and showed no trend in projected future primary production under RCP4.5 forcing and relatively weak trophic interactions arising from warming.

## Societal and policy implications

In common with many other regions worldwide, fisheries management decisions on total allowable catch (TAC) in the Barents Sea are based on annual assessments of species-by-species biomass and fishing mortality relative to reference points such as *B*_MSY_ and *F*_MSY_ (Hønneland 2014; ICES 2019). These are taken to be stable characteristics of each stock. The underlying assumption is that long-term average productivity is essentially constant. If this assumption becomes invalid, then the foundations of present fisheries management are undermined.

Progressive retreat of seasonal ice cover is having a transformational effect on primary production in the Barents Sea, and the balance between species is changing very rapidly (Fossheim et al. 2015). There is a high likelihood of significant trends in productivity throughout the food web, which poses a challenge not only to fisheries management, but to iconic Arctic fauna which are directly or indirectly affected by ice extent such as seabirds, cetaceans, pinnipeds and polar bears. There is a clear case for adopting an Ecosystem Approach to Fisheries (EAF) in this region as part of a strategy to manage the impacts of climate change on high trophic levels.

Pursuing an EAF requires data and modelling tools that span the food web and enable management strategy evaluation experiments to test the effects of proposed measures against reference points or targets defined not just for individual fish stock, but for the wider range of fauna. These tools need to work alongside, not instead of existing fisheries tools. The task is extremely challenging. Here, we have presented and demonstrated the potential of such a tool, which shows the scope for fisheries to affect the rest of the ecosystem against the backdrop of a changing physical environment, albeit at a coarse spatial and taxonomic resolution.

The societal implications of failing to move in the direction of an EAF could be particularly acute in the Arctic. Indigenous communities in these regions have subsisted on sustainable harvesting of marine fauna for generations. The threat to their way of life already posed by climate change could be accentuated without an EAF.

## Supplementary Information

Below is the link to the electronic supplementary material.

Supplementary file1 (PDF 875 kb)
