# Supplementary material for: Ecosystem approach to harvesting in the Arctic: Walking the tightrope between exploitation and conservation in the Barents Sea
Source: Ambio. 2021 Sep 3;51(2):456–70. doi: 10.1007/s13280-021-01616-9 (PMC8692644; doi:10.1007/s13280-021-01616-9)
Supplement: Supplementary file 1 — Supplementary file1 (PDF 875 kb) [file 13280_2021_1616_MOESM1_ESM.pdf]

Ambio

Electronic Supplementary Material
This supplementary material has not been peer reviewed.

Title: Ecosystem approach to harvesting in the Arctic: walking the tightrope between exploitation and conservation in the Barents Sea.

Authors: Michael R Heath, Déborah Benkort, Andrew S Brierley, Ute Daewel, Jack H. Laverick, Roland Proud, Douglas C Speirs

Appendix S1. Summary of ecological issues in the Barents Sea

The Barents Sea is a zone of transition between the Atlantic and the Arctic, and a region of rapid climate-related change. It is experiencing biogeochemical and biogeographical changes that are influencing community composition and energy-flow through food webs. South of the Polar Front the Barents Sea is free of ice all year, stratification is weak, and nutrients are available to support primary production in the euphotic zone. Further north, across the shelf-break and out into the deep Arctic basin, stratification due to the sea-ice freeze/melt cycle has historically limited nutrient supply (Tremblay and Gagnon 2009). As ice is lost, concomitant changes in underlying waters occur, and water column irradiance increases (Castellani et al. this volume, in review), further increasing primary production (Dalpadado et al. 2020).

Present predictions of changes in the upper trophic levels of the Barents Sea food web due to warming and ice loss are somewhat speculative based on extrapolation from historical data. As the influence of warmer, Atlantic waters extends from the south west, and Arctic waters recede northwards, zooplankton and fish community compositions have changed across the Barents Sea, with an increasing "borealisation" (Fossheim et al. 2015). Both bottom-up and top-down pressures can arise from these changes. For example, statistical modelling of historical data predicts that krill biomass in the Barents Sea increases in low-ice years but subsequently declines as higher trophic level populations respond (Stige et al. 2019). Earlier ice-melt may improve recruitment of the ice-associated forage fish *Boreogadus saida* (Polar cod) (Bouchard et al. 2017), and improved visibility in waters that are more brightly illuminated – along with changes in the size-structure of copepod communities (Berge et al. 2012) – will likely benefit planktivorous fish (Varpe et al. 2015). Predators of Polar cod, including seabirds and *Delphinapterus leucas* (beluga whale) may benefit from increasing prey abundance (Bouchard et al. 2017), as might *Pusa hispida* (ringed seal), although these seals depend on sea ice for breeding so increased prey will be countered by loss of habitat (Haug et al. 2017). Planktivorous whales may benefit from increased zooplankton production (Falk-Petersen et al. 2015), but polar bears (*Ursus maritimus*) will struggle as ice loss impacts their seal prey and their ability to hunt (Iversen et al. 2013).

References for Appendix S1

Berge, J., T.M. Gabrielsen, M. Moline and P.E. Renaud. 2012. Evolution of the Arctic Calanus complex: an Arctic marine avocado? *Journal of Plankton Research* 34(3): 191-195. doi:10.1093/plankt/fbr103.

Bouchard, C., M. Geoffroy, M. LeBlanc, A. Majewski, S. Gauthier, W. Walkusz, J. D. Reist and L. Fortier. 2017. Climate warming enhances polar cod recruitment, at least transiently. *Progress in Oceanography* 156: 121-129. doi:10.1016/j.pocean.2017.06.008.

Castellani, G., Veyssiere, G., Karcher, M., Stroeve, J., Banas, N.S., Bouman, H.A., Brierley, A.S. Connan, S., Cottier, F., Grosse, F., Hobbs, L., Katlein, C., Light, B., McKee, D., Orkney, A. and Proud, R. (in review). Shine a light: Underwater light and its ecological implications in a changing Arctic Ocean. *Ambio*.

Dalpadado, P., K.R. Arrigo, G.L. van Dijken, H.R. Skjoldal, E. Bagøien, A.V. Dolgov, I.P. Prokopchuk, and E. Sperfeld. 2020. Climate effects on temporal and spatial dynamics of phytoplankton and zooplankton in the Barents Sea. *Progress in Oceanography* 185, 102320. https://doi.org/10.1016/j.pocean.2020.102320

Falk-Petersen, S., V. Pavlov, J. Berge, F. Cottier, K. M. Kovacs and C. Lydersen. 2015. At the rainbow's end: high productivity fueled by winter upwelling along an Arctic shelf. Polar Biology 38(1): 5-11. doi:10.1007/s00300-014-1482-1

Fossheim, M., R. Primicerio, E. Johannesen, R. B. Ingvaldsen, M. M. Aschan and A.V. Dolgov. 2015. Recent warming leads to a rapid borealization of fish communities in the Arctic. *Nature Climate Change* 5(7): 673-+. doi:10.1038/nclimate2647.

Haug, T., B. Bogstad, M. Chierici, H. Gjosaeter, E.H. Hallfredsson, A.S. Hoines, A. Hakon-Hoel, R.B. Ingvaldsen, et al. 2017. Future harvest of living resources in the Arctic Ocean north of the Nordic and

Barents Seas: A review of possibilities and constraints. *Fisheries Research* 188: 38-57. doi:10.1016/j.fishres.2016.12.00.

Iversen, M., J. Aars, T. Haug, I. G. Alsos, C. Lydersen, L. Bachmann and K. M. Kovacs. 2013. The diet of polar bears (*Ursus maritimus*) from Svalbard, Norway, inferred from scat analysis. *Polar Biology* 36(4): 561-571. doi:10.1007/s00300-012-1284-2.

Tremblay, J. E. and J. Gagnon. 2009. The effects of irradiance and nutrient supply on the productivity of Arctic waters: a perspective on climate change. Influence of Climate Change on the Changing Arctic and Subarctic Conditions. *NATO Science for Peace and Security Series C: Environmental Security. Springer* 73-92. doi:10.1007/978-1-4020-9460-6_7

Varpe, O., M. Daase and T. Kristiansen. 2015. A fish-eye view on the new Arctic lightscape. *ICES Journal of Marine Science* 72(9): 2532-2538. doi:10.1093/icesjms/fsv129.

Appendix S2. Summary of ecosystem models of the Barents Sea

There have been numerous attempts to model food webs and ecosystem processes in the Barents Sea over the past twenty years. Dynamic multi-species population models, such as Gadget and its predecessors (Lindstrøm et al. 2009; and references therein), have been used to examine trophic interactions between small ensembles of key species (e.g. cod, herring, capelin, and minke whales), particularly to evaluate fishery management strategies. Such models have also been used to make predictions under climate change scenarios, although these are necessarily limited to assumed recruitment scenarios, or other directly imposed effects of temperature, food availability, or spatial overlap between key species (Howell and Bogstad 2010; Howell and Filin 2014).

By contrast, coupled physical/biogeochemical models represent the flow of nutrients through the entire ecosystem. These models link 3-dimensional general circulation models that capture the physical environment to a relatively small number of biological model components known as functional groups (e.g. diatoms, microzooplankton, and mesozooplankton) that represent the totality of trophically-similar species. Models covering the Barents Sea include SINMOD (Slagstad and McClimans 2005; Wassman et al 2006), HYCOM-NORWECOM (Samuelsen et al. 2015), and global models such as NEMO-MEDUSA (Popova et al. 2010, 2012, 2013). A 1-dimensional (vertical) version of the ECOSMO II model has been implemented for various stations in the Barents Sea (Benkort et al. 2020). Typically, physical-biological models do not represent trophic levels higher than zooplankton, and much of the effort in model validation is focussed on primary production (e.g. Babin et al. 2015).

End-to-end ecosystem models attempt to represent every trophic level. In order to achieve better representation at higher trophic levels, NORWECOM.E2E includes Individual-Based Models (IBMs) for particular species of zooplankton and fish that are fully coupled to the underlying physical-biological NORWECOM model. NORWECOM.E2E has been used to predict climate-driven changes in the distribution of the copepod *Calanus finmarchicus* (Skaret et al. 2014), and in evaluating acoustic/trawl survey designs for mackerel and herring (Holmin et al. 2020). Another end-to-end model that has been implemented for the Nordic and Barents Sea is Atlantis, a spatially structured model with physical forcing that represents the flow of nutrients through 53 functional groups and individual species (Hansen et al. 2016). Atlantis has been used to explore ecosystem perturbation and management and climate change scenarios (Hansen et al. 2019; Olsen et al. 2019; Nilsen et al. 2020). Several implementations of Ecopath-with-Ecosim (EwE) have also been developed and tuned to historical data (Blanchard et al. 2002; Skaret and Pitcher 2016; Bulgakova and Bobyrev 2020). EwE budgets biomass rather than conserving nutrients throughout the ecosystem, and these implementations are non-spatial, but as with Atlantis they represent numerous individual species explicitly.

When modelling the effects of climate change in the Arctic on ecosystem services it is essential to represent higher trophic levels, including charismatic megafauna such as marine mammals and seabirds as well as commercially exploited fish stocks. Equally, mass conservation is vital in order to link higher trophic levels to changes in biogeochemical fluxes, and spatial considerations with changing ice cover, for example, are also important. Nonetheless, many of the 'big picture' questions regarding sustainability can be addressed without needing to represent individual species or including all biogeochemical processes.

References for Appendix S2

Babin, M, S. Bélanger, I. Ellingsen, A. Forest, V. Le Fouest, T. Lacour, M. Ardyna, and D. Slagstad. 2015. Estimation of primary production in the Arctic Ocean using ocean colour remote sensing and coupled physical-biological models: Strengths, limitations and how they compare. *Progress in Oceanography* 139, 197-220. doi:10.1016/j.pocean.2015.08.008.

Benkort, D., U. Daewel, M. Heath, and C. Schrum. 2020. On the Role of Biogeochemical Coupling Between Sympagic and Pelagic Ecosystem Compartments for Primary and Secondary Production in the Barents Sea. *Frontiers in Environmental Science* 8:548013. doi:10.3389/fenvs.2020.548013.

Blanchard, J.L, J.K. Pinnegar, and S. Mackinson. 2002. *Exploring marine mammal-fishery interactions using 'Ecopath with Ecosim": Modelling the Barents Sea Ecosystem*. Cefas Science Series Technical Report 117.

Bulgova, T.I., and A.E. Bobyrev. 2020. The importance of Trophological Research in Multispecies Fishery Analysis. Biology Bulletin reviews 10, 19-27. doi:10.1134/S207908642001003X.

Hansen, C., Nash, R.D., K.F. Drinkwater, and S.S. Hjøllo. 2019. Management scenarios under climate change – A study of the Nordic and Barents Seas. *Frontiers in Marine Science* 6, 668. doi:10:3389/fmars.2019.00668.

Hansen, C., M. Skern-Mauritzen, G. van der Meeren, A. Jähkel, and K. Drinkwater. 2016. *Set-up of the Nordic and Barents Seas (NoBa) Atlantis Model*. Technical Report Number: 2-2016. Bergen: Norwegian Institute of Marine Research.

Holmin, A.J., E.A., Mousing, S.S. Hjøllo, M.D. Skogen, G. Huse, and N.O. Handegard. 2020. Evaluating acoustic-trawl survey strategies using an end-to-end ecosystem model. *ICES Journal of Marine Science* 77 2590-2599. doi:10.1093/icesjms/fsaal20.

Howell, D., and B. Bogstad. 2010. A combined Gadget/FLR model for management strategy evaluations of the Barents Sea fisheries. *ICES Journal of Marine Science* 67: 1998-2004. doi:10.1093/icesjms/fsq135.

Howell, D., and A.A. Filin. 2014. Modelling the likely impacts of climate-driven changes in cod-capelin overlap in the Barents Sea. *ICES Journal of Marine Science* 71: 72-80. doi:10.1093/icesjms/fst172.

Lindstrøm, U., S. Smout, D. Howell, and B. Bogstad. 2009. Modelling multispecies interactions in the Barents Sea ecosystem with special emphasis on minke whales, cod, herring and capelin. Deep Sea Research ii: Topical Studies in Oceanography 56, 2068-2079. doi:10.1016/j.dsr2.2008.11.017.

Nilsen, I., J. Kolding, C. Hansen, and D. Howell. 2020. Exploring balanced harvesting by using an Atlantis ecosystem model for the Nordic and Barents Seas. Frontiers in Marine Science 7, 70. doi:10.3389/fmars.2020.00070.

Olsen, E., C. Hansen, I. Nilsen, H. Perryman, and F. Vikebø. 2019. Ecological Effects and Ecosystem Shifts Caused by Mass Mortality Events on Early Life Stages of Fish. *Frontiers in Marine Science* 6, 669.doi:10.3389/fmars.2019.00669.

Popova, E.E., A. Yool, A.C. Coward, Y.K. Aksenov, S.G. Alderson, B.A. de Cuevas, and T.R. Anderson. 2010. Control of primary production in the Arctic by nutrients and light: insights from a high resolution ocean general circulation model. *Biogeosciences* 7, 3569–3591. doi:10.5194/bg-7-3569-2010.

Popova, E.E., A. Yool, A.C. Coward, F. Dupont, C. Deal, S. Elliott, E. Hunke, M.B. Jin, M. Steele, and J.L. Zhang. 2012. What controls primary production in the Arctic Ocean? Results from an intercomparison of five general circulation models with biogeochemistry. *Journal of Geophysical Research – Oceans* 117. doi:10.1029/2011JC007112.

Popova, E.E., A. Yool, Y. Aksenov, and A.C. Coward. 2013. Role of advection in Arctic Ocean lower trophic dynamics: a modeling perspective. *Journal of Geophysical Research – Oceans* 118, 1571–1586. doi:10.1002/jgrc.20126.

Samuelsen, A., C. Hansen, and H. Wehde. 2015. Tuning and assessment of the HYCOM-NORWECOM V2.1 biogeochemical modeling system for the North Atlantic and Arctic oceans. Geoscientific Model Development 8, 2187-2202. doi:10.5194/gmd-8-2187-2015.

Skaret, G., P. Dalpadado, S.S. Hjøllo, M.D. Skogen and E. Strand. 2014. *Calanus finmarchicus* abundance, production and population dynamics in the Barents Sea in a future climate. *Progress in Oceanography* 125 26-39. doi:10.1016/j.pocean.2014.04.008.

Skaret, G., and Pitcher, T.J. 2016. *An Ecopath With Ecosim Model of the Norwegian Sea and Barents Sea Validated Against Time Series of Abundance. 33 S.* Bergen: Norwegian Institute of Marine Research.

Slagstad, D., and T.A. McClimans. 2005. Modeling the ecosystem dynamics of the Barents Sea including the marginal ice zone: I. Physical and chemical oceanography. *Journal of Marine Systems* 58, 1–18. doi:0.1016/j.jmarsys.2005.05.005.

Wassmann, P., D. Slagstad, C.W. Riser, and M. Reigstad. 2006. Modelling the ecosystem dynamics of the Barents Sea including the marginal ice zone II. Carbon flux and interannual variability. *Journal of Marine Systems* 59, 1–24. doi:10.1016/jmarsys.2005.05.006.

Appendix S3. Internal and boundary driving data for the 2010s and 2040s versions of the StrathE2EPolar model.

Table S1. Details of the internal physical driving data inputs to StrathE2EPolar model. Climatological annual cycles of monthly averaged values for the 2010s and 2040s models are shown in Figure S1.

**Driving data**	**Units**	**Source**
Sea surface irradiance	$\mu E.m^{-2}.d^{-1}$	NEMO-MEDUSA input data
Sea temperature	°C	NEMO-MEDUSA output data
Air temperature	°C	NEMO-MEDUSA input data
Vertical diffusivity gradient	$m.d^{-1}$ (derived from the vertical diffusivity ($m^2.s^{-1}$) and mixing length scale (m))	NEMO-MEDUSA output data
Inshore significant wave height	m	CERA-20C 'Ocean Wave Synoptic Monthly Means' product accessed through ECMWF
Proportion of seabed disturbed	d^{-1} (aggregated over the three sediment classes in each zone)	Modelled from sediment grain size and tidal bed shear stress (Laverick et al. unpublished) (available for 2010s only, assumed to be identical in 2040s)
Suspended particulate matter	$g.m^{-3}$	Globcolour L3b; ftp://ftp.hermes.acri.fr/GLOB/merged/month/ (available for 2010s only, assumed to be identical in 2040s)
Ice-free area	proportion of whole model domain (*i.e.* ice cover * zonal area)	NEMO-MEDUSA output data
Ice-free %:	% of each zone ice free	NEMO-MEDUSA output data
Ice cover %:	Percent sea surface covered by ice in ice-affected areas	NEMO-MEDUSA output data
Ice thickness	m	NEMO-MEDUSA output data
Snow thickness	m	NEMO-MEDUSA output data

Table S2. Details of the external boundary driving data inputs to StrathE2EPolar model. Climatological annual cycles of monthly averaged values for the 2010s and 2040s models are shown in Figure S2.

**Driving data**	**Units**	**Source**
External inflows	m^3 per m^2 sea surface of model domain (derived from proportion input per layer volume, layer thicknesses and areas)	NEMO-MEDUSA output data
River discharge	m^3 per m^2 sea surface of model domain (derived from proportion input to inshore volume, and inshore layer thickness and area)	NEMO-MEDUSA input data
External boundary nitrate concentration	$mMN.m^{-3}$	NEMO-MEDUSA output data (total dissolved inorganic nitrogen disaggregated into nitrate and ammonia based on field observational data)
External boundary ammonia concentration	$mMN.m^{-3}$	NEMO-MEDUSA output data (total dissolved inorganic nitrogen disaggregated into nitrate and ammonia based on field observational data)
External boundary phytoplankton concentration	$mMN.m^{-3}$	NEMO-MEDUSA output data
External boundary detritus concentration	$mMN.m^{-3}$	NEMO-MEDUSA output data
River nitrate concentration	$mMN.m^{-3}$	Holmes et al. 2021 (available for 2010s only, assumed to be identical in 2040s)
River ammonia concentration	$mMN.m^{-3}$	Holmes et al. 2021 (available for 2010s only, assumed to be identical in 2040s)
River labile detritus concentration	$mMN.m^{-3}$	No data available
Atmospheric nitrate deposition flux	$mMN.m^{2}.d^{-1}$	EMEP data centre https://www.emep.int/mscw/mscw_moddata.html (available for 2010s only, assumed to be identical in 2040s)
Atmospheric ammonia deposition flux	$mMN.m^{2}.d^{-1}$	EMEP data centre https://www.emep.int/mscw/mscw_moddata.html (available for 2010s only, assumed to be identical in 2040s)

Reference for Table S2

Holmes, R.M., J.W. McClelland, S.E. Tank, R.G.M. Spencer, and A.I. Shiklomanov. 2021. Arctic Great Rivers Observatory. Water Quality Dataset, Version 2020-12-01. https://www.arcticgreatrivers.org/data (Web material).

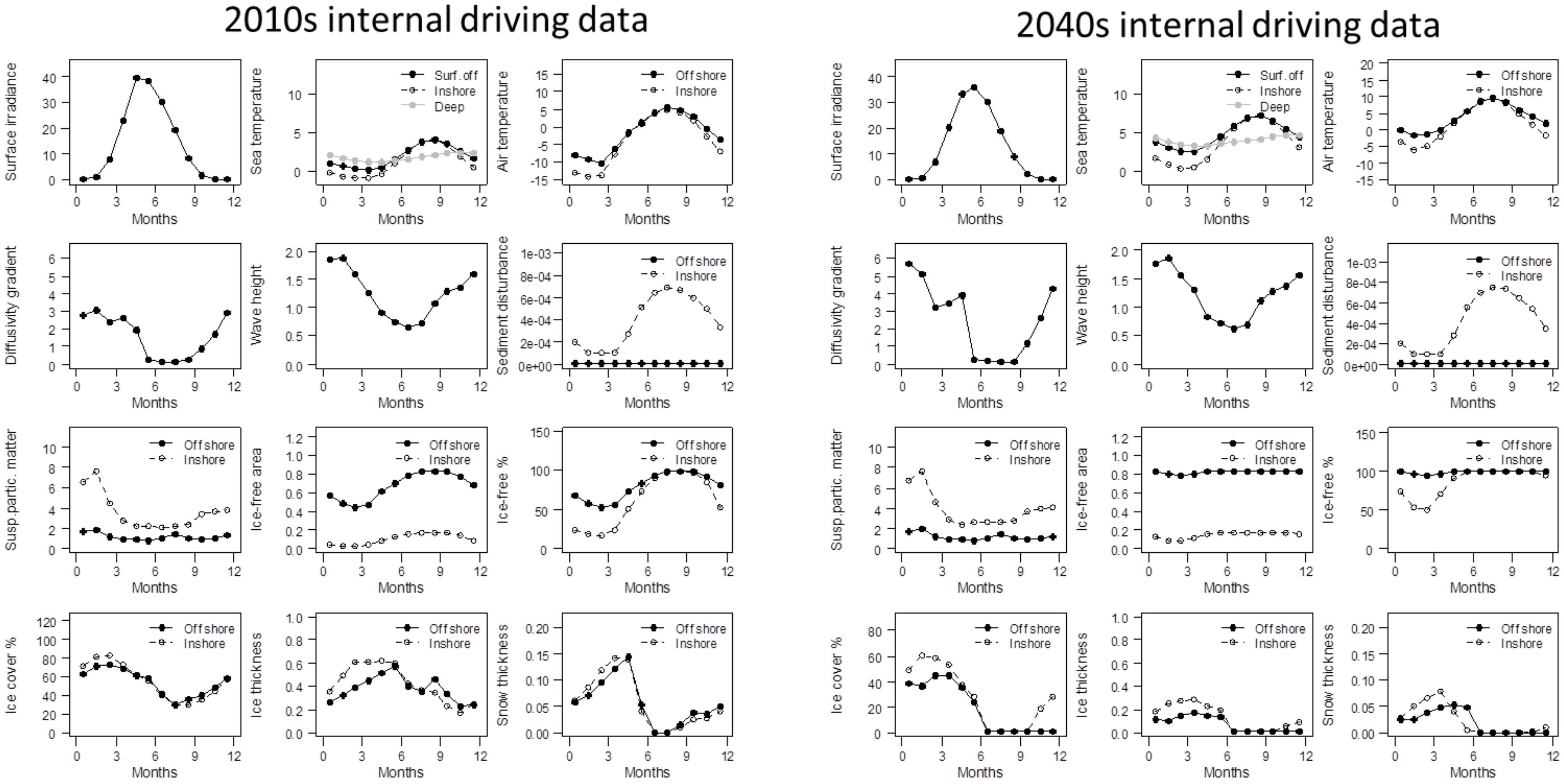

Figure S1. Climatological annual cycles of monthly averaged internal driving data for the 2010s and 2040s StrathE2EPolar models.

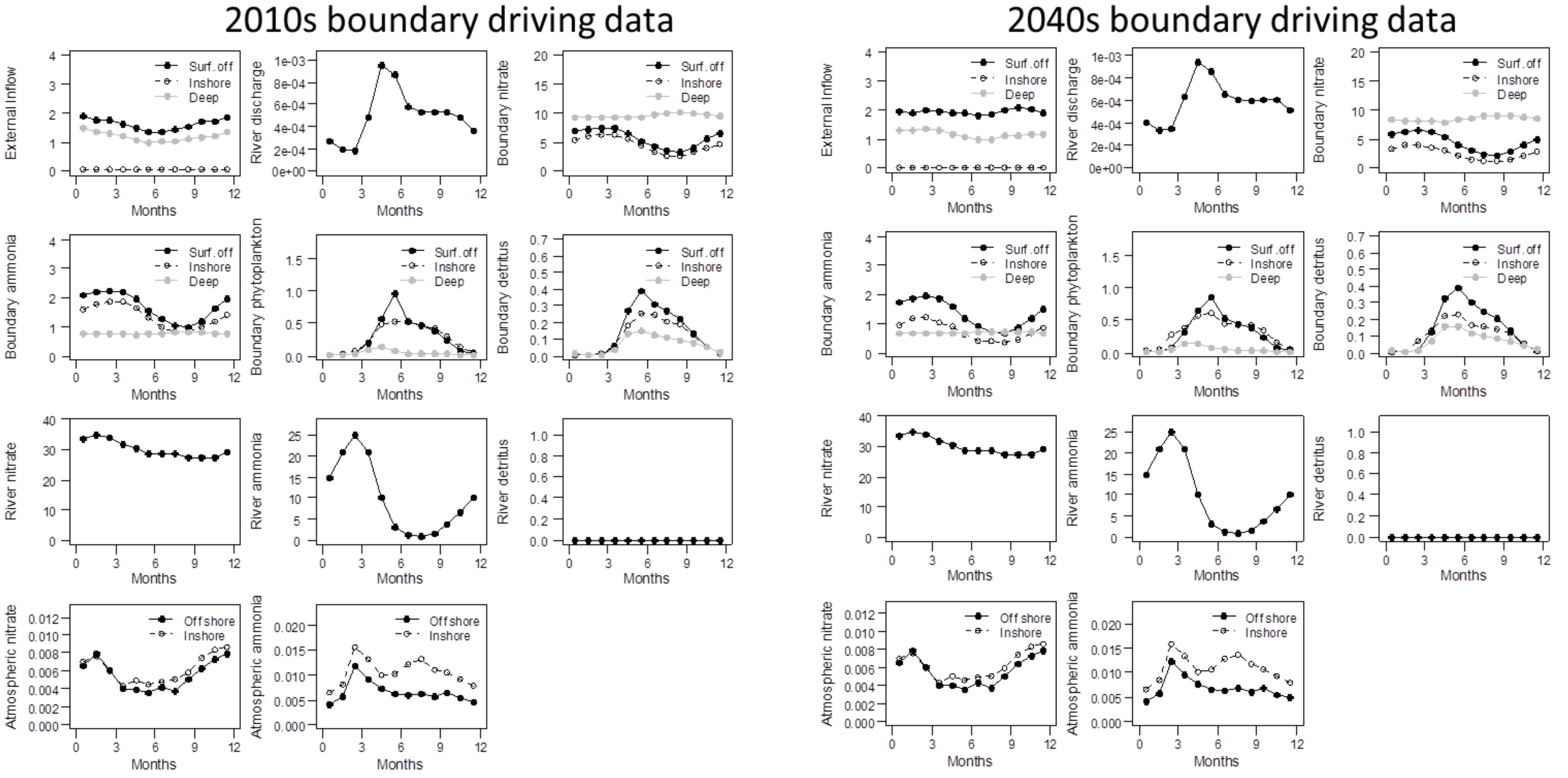

Figure S2. Climatological annual cycles of monthly averaged external boundary driving data for the 2010s and 2040s StrathE2EPolar models.

Appendix S4. Fishing gears represented in StrathE2EPolar

Table S3. Fishing gears represented in StrathE2EPolar and their selectivity for the various harvestable guilds in the model. Black cells indicate the main guild captured by each gear and grey cells show the various by-catch guilds. Guild code: PF = planktivorous fish,, DF = demersal fish, MF = migratory fish (mackerel), SDB -= suspension and deposit feeding benthos, CSB – carnivorous and scavenge feeding benthos, CZ = cantivorous zooplankton, BD – birds, PN = pinnipeds, CT = cetaceans, KP = macrophytes (kelp).

Gear category	PF	DF	MF	SDB	CSB	CZ	BD	PN	CT	KP
Pelagic trawls and seines										
Demersal otter trawl										
Demersal seine										
Demersal gillnets										
Longlines and jigging										
Recreational and subsistence fishing										
Shrimp trawls										
Creels										
Mollusc dredges										
Harpoons										
Rifles										
Kelp harvester										

Appendix S5. Observational data assembled as the target for optimization of the StrathE2EPolar, and comparison with the 'best-fit' model.

Table S4. Observational data assembled as the target for optimization of the StrathE2EPolar model by simulated annealing. The data are available as a csv file within the StrathE2EPolar R package. Also shown are the corresponding best-fit model values from the model. The overall likelihood of the observations given the model setup and driving data was 0.506.

Observed value	SD of observed value	Best-fit model value	Units	Description	Region	Time period	Source
868	43	843	mMN m^{-2} y^{-1}	Annual new primary production by phytoplankton	Barents Sea	2010-2018	ICES WGIBAR 2019 (189mgC m^{-2} d^{-1} annual average 2010-2018 converted to annual nitrogen units)
45.7	22.8	1.73	mMN m^{-2} y^{-1}	Annual total primary production by ice algae	Barents Sea	2010-2018	Benkort et al. 2020
320	128	284	gC m^{-2} y^{-1}	Annual within forest net production of macrophytes	Northern Norway	General	Sjøtun et al. 1995, Dommasnes et al. 2001
396	158	844	mMN m^{-2} y^{-1}	Annual ominivorous zooplankton gross production	Barents Sea	1990-1995	Blanchard et al.. 2002
60.9	24.4	21.4	mMN m^{-2} y^{-1}	Annual carnivorous zooplankton gross production	Barents Sea	1990-1995	Blanchard et al. 2002
3.18	1.27	2.24	mMN m^{-2} y^{-1}	Annual planktivorous fish gross production	Barents Sea	2011-2017	Dommasnes et al 2001 and data from Protozorkevich, and van der Meeren 2020.
2.64	1.06	3.72	mMN m^{-2} y^{-1}	Annual demersal fish gross production	Barents Sea	2011-2017	Dommasnes et al 2001 and data from Protozorkevich, and van der Meeren 2020.
386	154	434	mMN m^{-2} y^{-1}	Annual susp/dep benthos gross production	Barents Sea	1990-1995	Blanchard et al ..2002
35.4	14.1	19.5	mMN m^{-2} y^{-1}	Annual carn/scan benthos gross production	Barents Sea	1990-1995	Blanchard et al 2002
0.0126	5.04E-03	0.0042	mMN m^{-2} y^{-1}	Annual net production of birds	Barents Sea	1997	Dommasnes et al. 2001
0.0153	6.14E-03	0.00899	mMN m^{-2} y^{-1}	Annual net production of pinnipeds	Barents Sea	1997	Dommasnes et al. 2001
0.0202	8.08E-03	0.0148	mMN m^{-2} y^{-1}	Annual net production of cetaceans	Barents Sea	1997	Dommasnes et al 2001
1.22E-04	4.88E-05	0.00016	mMN m^{-2} y^{-1}	Annual net production of maritime mammals	Barents Sea	2000-2009	Kovacs et al. 2009, Suprenand et al 2018

2.15	0.86	0.1287	mMN m^{-2} y^{-1}	Annual consumption of planktivorous fish by fish	Barents Sea	1997	Dommasnes et al. 2001
1.500	0.600	0.06088	mMN m^{-2} y^{-1}	Annual consumption of demersal fish by fish	Barents Sea	1997	Dommasnes et al. 2001
14.8	5.9	15.32	mMN m^{-2} y^{-1}	Annual consumption of omnivorous zooplankton by fish and fish larvae	Barents Sea	1997	Dommasnes et al 2001
51.8	20.7	62.60	mMN m^{-2} y^{-1}	Annual consumption of omniv zooplankton by carn zooplankton	Barents Sea	1990-1995	Blanchard et al 2002
3.26	1.30	3.797	mMN m^{-2} y^{-1}	Annual consumption of benthos by fish	Barents Sea	1997	Dommasnes et al 2001
1.38	0.55	0.029	mMN m^{-2} y^{-1}	Annual food consumption by birds	Barents Sea	1995-2005	Gabrielsen 2009
0.77	0.31	0.668	dimensionless	Proportion planktivorous fish in diet of birds	Barents Sea	1997	Dommasnes et al 2001
0.04	0.016	0.0145	dimensionless	Proportion demersal fish in diet of birds	Barents Sea	1997	Dommasnes et al 2001
0.045	0.018	0.00511	dimensionless	Proportion migratory fish in diet of birds	Barents Sea	1997	Dommasnes et al 2001
0.001	0.0004	0.00101	dimensionless	Proportion discards in diet of birds	Barents Sea	1997	Dommasnes et al 2001
3.18	1.27	0.0616	mMN m^{-2} y^{-1}	Annual food consumption by pinnipeds	Barents Sea	1997	Dommasnes et al 2001
0.40	0.16	0.545	dimensionless	Proportion planktivorous fish in diet of pinnipeds	Barents Sea	1997	Dommasnes et al 2001
0.098	0.039	0.0544	dimensionless	Proportion demersal fish in diet of pinnipeds	Barents Sea	1997	Dommasnes et al 2001
0.011	0.0044	0.00064	dimensionless	Proportion migratory fish in diet of pinnipeds	Barents Sea	1997	Dommasnes et al 2001
3.040	1.22	0.10881	mMN m^{-2} y^{-1}	Annual food consumption by cetaceans	Barents Sea	1997	Dommasnes et al 2001
0.254	0.102	0.453	dimensionless	Proportion planktivorous fish in diet of cetaceans	Barents Sea	1997	Dommasnes et al 2001
0.109	0.043	0.0782	dimensionless	Proportion demersal fish in diet of cetaceans	Barents Sea	1997	Dommasnes et al 2001
0.117	0.047	0.0114	dimensionless	Proportion migratory fish in diet of cetaceans	Barents Sea	1997	Dommasnes et al 2001
0.502	0.201	0.457	dimensionless	Proportion omni and carn zoo in diet of cetaceans	Barents Sea	1997	Dommasnes et al 2001
2.47E-03	0.001	0.0010	mMN/m2/y	Annual food consumption by maritime mammals	Barents Sea	2000-2009	Kovacs et al 2009, Suprenand et al 2018

0.750	0.300	0.855	dimensionless	Proportion pinnipeds in diet of maritime mammals	Barents Sea	2000-2009	Kovacs et al 2009, Suprenand et al 2018
0.100	0.040	0.129	dimensionless	Proportion cetaceans in diet of maritime mammals	Barents Sea	2000-2009	Kovacs et al 2009, Suprenand et al 2018
0.175	0.0116	0.169	mMN m^{-2} y^{-1}	Annual planktivorous fish landings (live weight)	Barents Sea	2011-2019	Norwegian Fisheries Directorate, STECF, ICES/FAO
0.323	0.032	0.330	mMN m^{-2} y^{-1}	Annual demersal fish landings (live weight)	Barents Sea	2011-2019	Norwegian Fisheries Directorate, STECF, ICES/FAO
0.0016	0.0002	0.0016	mMN m^{-2} y^{-1}	Annual migratory fish landings (live weight)	Barents Sea	2011-2019	Norwegian Fisheries Directorate, STECF, ICES/FAO
2.18E-07	2.18E-08	2.10E-07	mMN m^{-2} y^{-1}	Annual susp/dep benthos landings (live weight)	Barents Sea	2011-2019	Norwegian Fisheries Directorate, STECF, ICES/FAO
0.015	0.0015	0.0160	mMN m^{-2} y^{-1}	Annual carn/scav benthos landings (live weight)	Barents Sea	2011-2019	Norwegian Fisheries Directorate, STECF, ICES/FAO
1.56E-07	1.56E-08	1.57E-07	mMN m^{-2} y^{-1}	Annual carn zooplankton landings (live weight)	Barents Sea	2011-2019	Norwegian Fisheries Directorate, STECF, ICES/FAO
8.60E-05	3.44E-05	7.70E-05	mMN m^{-2} y^{-1}	Annual pinniped landings by seal hunters	Barents Sea	2011-2019	Norwegian Fisheries Directorate, STECF, ICES/FAO
9.06E-04	3.62E-04	0.00092	mMN m^{-2} y^{-1}	Annual cetacean landings by whale hunters	Barents Sea	2011-2019	Norwegian Fisheries Directorate, STECF, ICES/FAO
0.650	0.260	0.787	y^{-1}	Annual CARBON gross PB ratio of macrophytes	Northern Norway	General	Sjøtun et al 1995, Dommasnes et al 2001
10.0	4.0	7.75	y^{-1}	Annual gross PB ratio larvae of susp/dep benthos	General value	General	Mackinson and Daskalov 2007
10.0	4.0	8.84	y^{-1}	Annual gross PB ratio larvae of carn/scav benthos	General value	General	Mackinson and Daskalov 2007
6.44	2.58	8.53	y^{-1}	Annual gross PB ratio susp/dep feeding benthos	Barents Sea	1990-1995	Blanchard et al 2002
2.42	0.97	2.221	y^{-1}	Annual gross PB ratio carn/scav feeding benthos	Barents Sea	1990-1995	Blanchard et al 2002
10.0	4.0	8.83	y^{-1}	Annual gross PB ratio omniv zooplankton	Barents and Norwegian Sea	1997	Dommasnes et al 2001
2.814	1.126	2.293	y^{-1}	Annual gross PB ratio carniv zooplankton	Barents and Norwegian Sea	1997	Dommasnes et al 2001
4.0	1.6	0.979	y^{-1}	Annual gross PB ratio larvae of planktivorous fish	General value	General	Mackinson and Daskalov 2007
4.0	1.6	3.762	y^{-1}	Annual gross PB ratio larvae of demersal fish	General value	General	Mackinson and Daskalov 2007
0.640	0.256	0.601	y^{-1}	Annual gross PB ratio planktivorous fish	Barents and Norwegian Sea	1997	Dommasnes et al 2001
1.049	0.420	0.147	y^{-1}	Annual gross PB ratio demersal fish	Barents and Norwegian Sea	1997	Dommasnes et al 2001

0.600	0.240	0.332	y^{-1}	Annual gross PB ratio migratory fish	Barents and Norwegian Sea	1997	Dommasnes et al 2001
1.00	0.40	0.338	y^{-1}	Annual net PB ratio birds	Barents and Norwegian Sea	1997	Dommasnes et al 2001
0.07	0.028	0.124	y^{-1}	Annual net PB ratio pinnipeds	Barents and Norwegian Sea	1997	Dommasnes et al 2001
0.04	0.016	0.0326	y^{-1}	Annual net PB ratio cetaceans	Barents and Norwegian Sea	1997	Dommasnes et al 2001
0.15	0.060	0.0591	y^{-1}	Annual net PB ratio maritime mammals	Beaufort Sea	General	Suprenand et al 2018
0.30	0.10	0.316	dimensionless	Annual average proportion of macrophyte C uptake which is exuded	General value	General	Abdullah and Fredriksen 2004
0.12	0.20	0.116	dimensionless	Annual average molar NC ratio of macrophytes	General value	General	Sjotun et al 1996, Broch and Slagstad 2012
90.7	36.3	87.1	mMN m^{-2} y^{-1}	Annual water and sediment denitrification	Chuchi Sea	2013	McTigue et al 2016
0.0320	0.0033	0.0316	dimensionless	Proportion of demersal fish catch discarded	Barents Sea	2011-2019	Norwegian Fisheries Directorate, STECF, ICES/FAO
1.90	0.54	2.01	mMN m^{-3}	Feb Mar average ammonia conc in sea ice when present	Fram Strait	General	Thomas et al 1995
18.6	10.2	20.8	mMN m^{-3}	Annual average ammonia conc in porewater of mud gs 0.12mm	Barents Sea	2017	Freitas et al 2020
0.903	0.287	0.804	mMN m^{-3}	Feb Mar average nitrate conc in sea ice when present	Fram Strait	General	Thomas et al 1995
4.02	1.44	3.183	mMN m^{-3}	Annual average nitrate conc in porewater of mud gs 0.12mm	Barents Sea	2017	Freitas et al 2020
0.0353	0.00715	0.0318	% gN (g dry $sed)^{-1}$	Annual average organic N content of sand gs 0.25mm (0.19-0.43mm)	Barents Sea	General	Laverick et al. submitted
0.1360	0.0272	0.121	% gN (g dry $sed)^{-1}$	Annual average organic N content of mud gs 0.12mm (0.03-0.07mm)	Barents Sea	General	Laverick et al. submitted
5.50	0.764	6.649	mMN m^{-3}	Average winter (Nov-Feb) nitrate conc shallow layer	Barents Sea	2010-2019	BODC, ICES and Norwegian Polar Institute data centres
3.06	2.08	1.586	mMN m^{-3}	Average summer (May-Aug) nitrate conc shallow layer	Barents Sea	2010-2019	BODC, ICES and Norwegian Polar Institute data centres
9.64	2.11	7.459	mMN m^{-3}	Average summer (May-Aug) nitrate conc deep layer	Barents Sea	2010-2019	BODC, ICES and Norwegian Polar Institute data centres
0.216	0.264	0.456	mMN m^{-2}	Average summer (April-Sept) chlorophyll in offshore sea ice	Offshore ice Arctic	General	Melinkov et al. 2002, Thomas et al.1995, Schunemann 2004, Ehrlich et al. 2020,

							Lange et al.2016, Gradinger and Zhang 1997; assuming_Redfield_and_ carbon:chlorophyll = 20 $g.g^{-1}$
3.099	1.525	2.231	mMN m^{-2}	Average summer (April-Sept) chlorophyll in inshore sea ice	Landfast ice Arctic	General	Appolonio 1965, Lange et al. 2015, Campbell et al. 2015, Schunemann 2004; assuming_Redfield_and_ carbon:chlorophyll = 20 $g.g^{-1}$
1.33E-05	5.33E-06	1.39E-05	mMN m^{-2} y^{-1}	Annual bycatch of birds	Barents Sea	2000-2019	Synthesis of literature data
9.70E-06	3.88E-06	1.10E-05	mMN m^{-2} y^{-1}	Annual bycatch of pinnipeds	Barents Sea	2006-2014	Synthesis of literature data
2.38E-05	9.53E-06	2.41E-05	mMN m^{-2} y^{-1}	Annual bycatch of cetaceans	Barents Sea	2000-2019	Synthesis of literature data
0.15	0.05	0.147	dimensionless	Proportion of macrophyte annual nitrogen uptake exported as beachcast	New Zealand	General	Zemke White et al 2005

References for Table S4

Abdullah, M.I. & Fredriksen, S. (2004). Production, respiration and exudation of dissolved organic matter by the kelp *Laminaria hyperborea* along the west coast of Norway. *Journal of the Marine Biological Association of the UK*, 84, 887-894.

Appolonio, S. 1965. Chlorophyll in Arctic sea ice. *Arctic: Short Papers and Notes* 18: 118-122. https://doi.org/10.14430/arctic3457

Benkort, D., U. Daewel, M. Heath, and C. Schrum. 2020. On the Role of Biogeochemical Coupling Between Sympagic and Pelagic Ecosystem Compartments for Primary and Secondary Production in the Barents Sea. *Frontiers in Environmental Science* 8:548013. doi:10.3389/fenvs.2020.548013.

Blanchard, J.L, J.K. Pinnegar, and S. Mackinson. 2002. *Exploring marine mammal-fishery interactions using 'Ecopath with Ecosim": Modelling the Barents Sea Ecosystem*. Cefas Science Series Technical Report 117.

Broch, O.J. & Slagstad, D. (2012). Modelling seasonal growth and composition of the kelp *Saccharina latissimi*. Journal of. Applied Phycology 24, 759-776.

Campbell, K., C.J. Mundy, D.G. Barber, and M. Gosselin. 2015. Characterizing the sea ice algae chlorophyll a–snow depth relationship over Arctic spring melt using transmitted irradiance. *Journal of Marine Systems* 147: 76–84.

Dommasnes, A., Christensen, V., Ellertsen,B., Kvamme, C., Melle, W., Nøttestad, L., Pedersen, T., Tjelmeland, S. and Zeller, D. 2001. An Ecopath model for the Norwegian and Barents Sea. In: S. Guenette, V. Christensen, D. Pauly (Eds.), Fisheries Impact on North Atlantic Ecosystems: Models and Analyses, Fisheries Centre Research Reports 9(3). University of British Colombia, Vancouver (2001), pp. 213-240

Ehrlich, J., L. F.L. Schaafsma, B.A. Bluhm, I. Peeken, G. Castellani, A. Brandt, and H. Flores. 2020. Sympagic fauna in and under Arctic pack ice in the annual sea-ice system of the New Arctic. *Frontiers in Marine Science* 7:452. doi: 10.3389/fmars.2020.00452

Freitas FS, Hendry KR, Henley SF, Faust JC, Tessin AC, Stevenson MA, Abbott GD, März C, Arndt S. 2020. Benthic-pelagic coupling in the Barents Sea: an integrated data-model framework. *Phil. Trans. R. Soc. A* 378: 20190359. http://dx.doi.org/10.1098/rsta.2019.0359Freitas et al 2020

Gabrielsen, G.W. 2009. Seabirds in the Barents Sea. . In Sakshaug, E., Johnsen, G.H, and Kovacs, K.M. Ecosystem *Barents Sea* Chapter 17, 4.5-*452*. Tapir Academic Press.

Gradinger R, Q. Zhang. 1997. Vertical distribution of bacteria in Arctic sea ice from the Barents and Laptev Seas. *Polar Biology* 17: 448–454.

ICES. 2019. The Working Group on the Integrated Assessments of the Barents Sea (WGIBAR). ICES Scientific Reports. 1:42. 157 pp. http://doi.org/10.17895/ices.pub.5536

Kovacs, K.M., Haug, T. and Lydersen, C. 2009. Marine mammals of the Barents Sea. In Sakshaug, E., Johnsen, G.H, and Kovacs, K.M. Ecosystem *Barents Sea* Chapter 18, *453-496*. Tapir Academic Press.

Lange, B.A., C. Michel, J.F. Beckers, J.A. Casey, H. Flores, I. Hatam, et al. 2015. Comparing springtime ice-algal chlorophyll *a* and physical properties of multi-year and first-year sea ice from the Lincoln Sea. *PLoS ONE* 10(4): e0122418. doi:10.1371/journal.pone.0122418

Lange, B. A., C. Katlein, M. Nicolaus, I. Peeken, and H. Flores. 2016. Sea ice algae chlorophyll a concentrations derived from under-ice spectral radiation profiling platforms, *Journal of Geophysical Research, Oceans* 121: 8511–8534. doi:10.1002/2016JC011991.

Laverick, J., Speirs, D.C. and Heath, M.R. submitted. Synthetic shelf sediment maps for the Greenland Sea and Barents Sea. Earth Systems Science Data.

Mackinson, S., Daskalov, G., 2007. An ecosystem model of the North Sea to support an ecosystem approach to fisheries management: description and parameterisation. Scientific Series Technical Report, Cefas Lowestoft, 142, 195pp.

McTigue, N. D. Gardner, W.S., Dunton, K,H. and Hardison, A.K. l.2016. Biotic and abiotic controls on co-occurring

nitrogen cycling processes in shallow Arctic shelf sediments. Nature Communications. 7, 13145. doi: 10.1038/ncomms13145 (2016).

Melnikov, I.A., E.G. Kolosova, H.E. Welch, and L.S. Zhitina. 2002. Sea ice biological communities and nutrient dynamics in the Canada Basin of the Arctic Ocean. *Deep-Sea Research I* 49: 1623–1649.

Protozorkevich, D. and van der Meeren, G.I. (eds) 2020.Survey report from the joint Norwegian/ Russian ecosystem survey in the Barents Sea and adjacent waters August-October 2019. IMR/PINRO Joint Report Series, 1-2020, 93pp

Schunemann, ,M. 2004. Studies on the Arctic pack-ice habitat and sympagic meiofauna – seasonal and regional variabilities. PhD Thesis, University of Kiel. 103 pp.

Sjøtun, K., Fredriksen, S., Rueness, J. and Lein, T. E. 1995. Ecological studies of the kelp *Laminaria hyperborea* (Gunnerus) Foslie in Norway. In Ecology of Fjords and Coastal Waters, pp 525-536. Ed. by H. R. Skjoldal, C. C. E. Hopkins, K. E. Erikstad and H. P. Leinaas. Elsevier Science B. V., Amsterdam.

Sjøtun, K., Fredriksen, S. & Rueness, J. (1996). Seasonal growth and carbon and nitrogen content in canopy and first-year plants of *Laminaria hyperborea* (Laminariales, Phaeophyceae). *Phycologia* 35, 1-8.

Suprenand, Paul M.; Ainsworth, Cameron H.; and Hoover, Carie, "Ecosystem Model of the Entire Beaufort Sea Marine Ecosystem: A Temporal Tool for Assessing Food-Web Structure and Marine Animal Populations from 1970 to 2014" (2018).*Marine Science Faculty Publications*. 261.

Thomas, D.N., Lara, R.J., Eicken, H., Kattner, G. and Skoog, A. 1995. Dissolved organic matter in Arctic multi-year sea ice during winter: major components and relationship to ice characteristics. Polar Biology 15, 477-483

Zemke-White, W.L., Speed, S.R. & McClary, D.J. (2005). Beach-cast seaweed: a review. *New Zealand Fisheries Assessment Report* 2005/44. 47pp

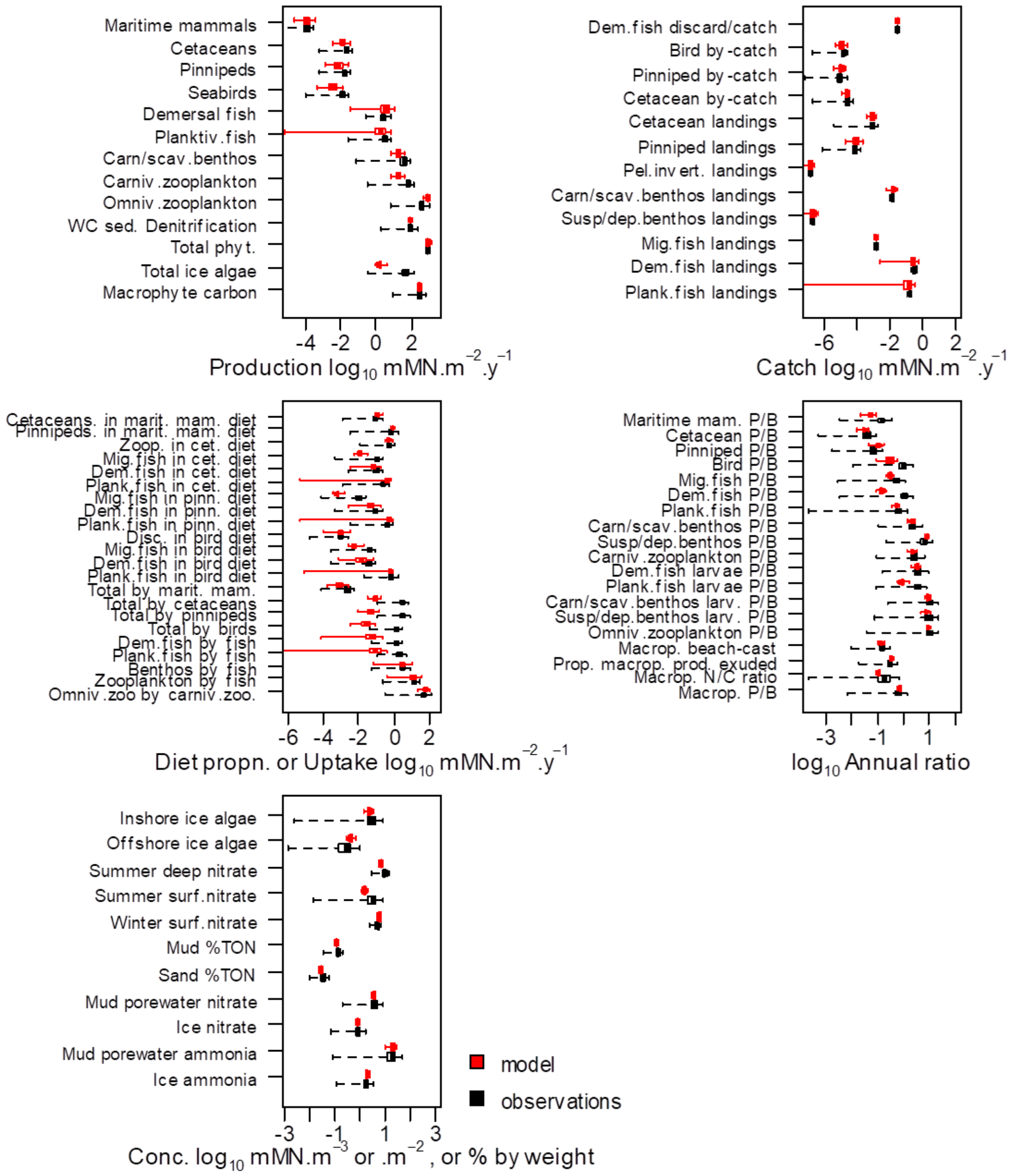

Figure S3. Comparison between the 'best fit' 2010s StrathE2EPolar model results, and the observational target data used for computational parameter optimization. Red box and whiskers are the modal results and their credible intervals derived from likelihood weighted Monte Carlo analysis, black are the observational target data and their standard deviations (uncertainties). Boxes for each measure represent the inter-quartile ranges, whiskers span 0.5% – 99.5% intervals.

Appendix S6. Methods for acoustic data processing

Raw data were read using the pyEcholab Python (version 3.7) package (https://github.com/CI-CMG/pyEcholab) and processed using the Echopy Python package (https://github.com/open-ocean-sounding/echopy). Processing included identifying seabed (and removing detections at and below it), subtracting background noise, removing pings that were attenuated or that included transient noise, masking impulse noise (interference from other sound sources) and removing data that contained false bottom echoes (Ryan et al. 2015). Nautical-Area Scattering Coefficient (NASC, m^2 nmi^{-2}: average received echo energy over a given depth range scaled up to a square nautical mile) values were computed for the remaining data averaged over 1 km along track intervals (station data were not analysed) between 15 m (maximum vessel draft plus echosounder near-field for all surveys, vessels and frequencies) and 400 m (lower extent of model domain).

NASC values at 18 kHz were used as a proxy for fish biomass. At this frequency the contribution of smaller scatterers such as zooplankton is expected to be < 1%. Fish, on the other hand, which have gas-filled swim bladders, are very strong acoustic targets and will likely make up the majority of the received echo energy at this frequency (Proud et al. 2019). NASC values at 120 kHz and where NASC 120 kHz > 38 kHz > 18 kHz (i.e. Rayleigh scattering from small targets such as zooplankton) were used as a proxy for macro-zooplankton biomass (similar methods are used to discriminate Antarctic krill/zooplankton from fish in the Southern Ocean, see Brierley et al. 1998).

References for Appendix S6

Brierley, A.S., P. Ward, J.L. Watkins, and C. Goss. 1998. Acoustic discrimination of Southern Ocean zooplankton. *Deep Sea Research Part II: Topical Studies in Oceanography* 45: 1155–1173. https://doi.org/10.1016/S0967-0645(98)00025-3

Proud, R., N.O. Handegard, R.J. Kloser, M.J. Cox, and A.S. Brierley. 2019. From siphonophores to deep scattering layers: uncertainty ranges for the estimation of global mesopelagic fish biomass. *ICES Journal of Marine Science* 76: 718–733. https://doi.org/10.1093/icesjms/fsy037

Ryan, T. E., R.A. Downie, R.J. Kloser, and G. Keith. 2015. Reducing bias due to noise and attenuation in open-ocean echo integration data. *ICES Journal of Marine Science* 72: 2482–2493. doi:10.1093/icesjms/fsv121.

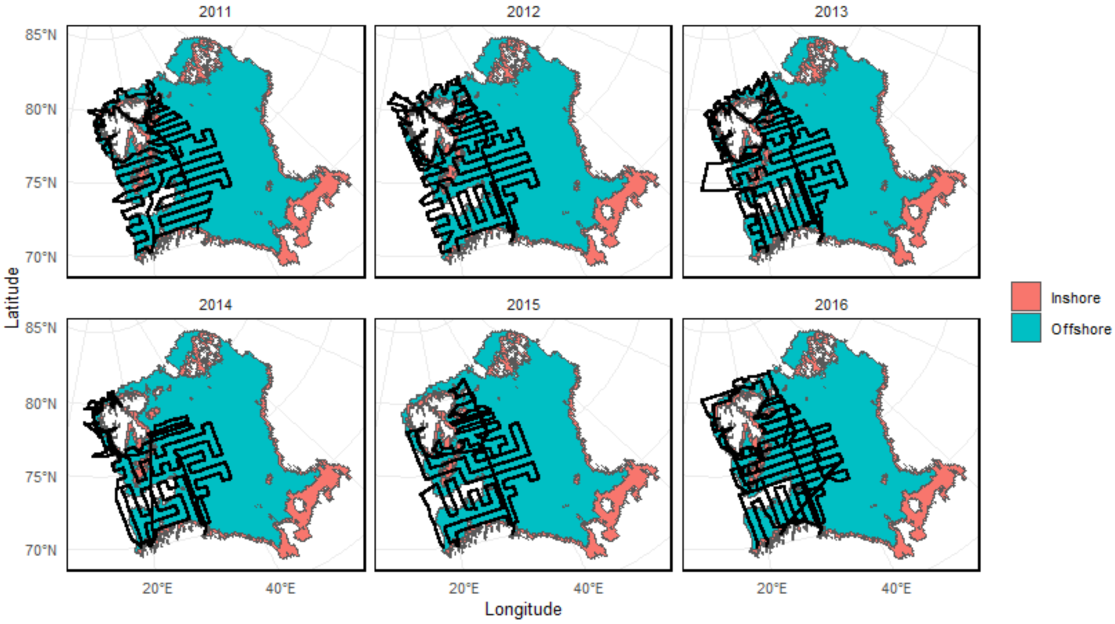

Figure S4. Barents Sea Ecosystem Survey tracks (2011-2016) overlaid onto the inshore and offshore zones of the StrathE2EPolar model.
